# Depressive symptoms and HIV risk behaviours among adolescents enrolled in the HPTN071 (PopART) trial in Zambia and South Africa

**DOI:** 10.1371/journal.pone.0278291

**Published:** 2022-12-01

**Authors:** Kwame Shanaube, Thomas Gachie, Graeme Hoddinott, Albertus Schaap, Sian Floyd, Tila Mainga, Virginia Bond, Richard Hayes, Sarah Fidler, Helen Ayles, on behalf of the HPTN071 (PopART) Study Team

**Affiliations:** 1 Zambart, Lusaka, Zambia; 2 London School of Hygiene and Tropical Medicine, London, United Kingdom; 3 Desmond Tutu TB Centre, Department of Paediatrics and Child Health, Faculty of Medicine and Health Sciences, Stellenbosch University, Cape Town, South Africa; 4 Department of infectious disease, Imperial College, London, Imperial College NIHR BRC, United Kingdom; Emory University School of Medicine, UNITED STATES

## Abstract

**Background:**

Mental health is a critical and neglected public health problem for adolescents in sub-Saharan Africa. In this paper we aim to determine the prevalence of depressive symptoms and the association with HIV risk behaviours in adolescents aged 15–19 years in Zambia and SA.

**Methods:**

We conducted a cross-sectional survey from August-November 2017 in seven control communities of HPTN 071 (PopART) trial (a community-randomised trial of universal HIV testing and treatment), enrolling approximately 1400 eligible adolescents. HIV-status was self-reported. Depressive symptoms were measured with the Short Mood and Feelings Questionnaire (SMFQ), with a positive screen if adolescents scored ≥12. We fitted a logistic regression model to identify correlates of depressive symptoms with subgroup analyses among those who self-reported ever having had sex, by gender and country.

**Results:**

Out of 6997 households approached, 6057 (86.6%) were enumerated. 2546 adolescents were enumerated of whom 2120 (83.3%) consented to participate and were administered the SMFQ. The prevalence of depressive symptoms was 584/2120 (27.6%) [95%CI: 25.7%-29.5%]. Adolescents in SA were less likely to experience depressive symptoms (Adjusted Odds Ratio [AOR] = 0.63 (95% CI: 0.50, 0.79), p-value<0.0001).

Female adolescents (AOR = 1.46 (95% CI: 1.19, 1.81), p-value<0.0001); those who reported ever having sex and being forced into sex (AOR = 1.80 (95% CI: 1.45, 2.23), p-value<0.001) and AOR = 1.67 (95% CI: 0.99, 2.84); p-value = 0.057 respectively) were more likely to experience depressive symptoms. Among 850 (40.1%) adolescents who self-reported to ever having had sex; those who used alcohol/drugs during their last sexual encounter were more likely to experience depressive symptoms (AOR = 2.18 (95% CI: 1.37, 3.47); p-value = 0.001), whereas those who reported using a condom were less likely to experience depressive symptoms (AOR = 0.74 (95% CI: 0.55, 1.00); p-value = 0.053).

**Conclusion:**

The prevalence of depressive symptoms among adolescents ranged from 25–30% and was associated with increased HIV-risk behaviour.

## Introduction

Mental health disorders (MHDs) are a critical and neglected public health problem for adolescents aged 10–19 years in sub-Saharan Africa (SSA) [1]. MHDs account for 16% of the global burden of disease and injury in adolescents [2], with half of all MHDs starting by 14 years of age and most being undetected and untreated [3]. These conditions often persist for a long time, severely disrupting adolescents’ access to livelihoods, health care, and education, and exposing them to stigma, isolation, suicidal behaviour, discrimination, and sexual abuse [4–6]. Adolescents’s MHDs also extend to adulthood, limiting opportunities to lead fulfilling lives as adults.

Depression is one of the most common MHDs among adolescents globally [6–8]. A systematic review covering general population studies encompassing 14 409 adolescents from 16 different sub-Sahara Africa (SSA) countries found a prevalence of depression of 26.9% (IQR 20.1–31.1) [9]. Another review on the prevalence of MHDs in SSA adolescents found that one in seven children and adolescents (14.3%) experience significant psychological challenges, and one in ten (9.5%) qualifies for a psychiatric diagnosis [7]. Depression can be attributed to physical, biological, emotional and social changes that form part of adolescent formative period of transition [1, 10]. A systematic review of global MHDs among adolescents showed that risk factors can be categorized into life-long risk factors, such as genetic background, and age-specific risk factors such as substance use, developmental-behavioural disorders such as stress of puberty, and cognitive changes [11].

Research linking HIV-risk behaviour and specific MHDs problems has yielded mixed results. Some studies found no associations between specific diagnoses and HIV-risk behaviours, but other findings support such links. Internalizing problems (low self-esteem, depression, anxiety) are associated with low perceived self-efficacy, decreased assertiveness, and minimal ability to negotiate safe sex with a partner [12, 13]. Depression is also linked to illicit drug use, sexually permissive attitudes, having sexually active friends, sexual behaviour, low contraception use and high risk of pregnancy [14, 15]. Moreover, hopelessness and helplessness may reduce adolescents’ motivation to make health-promoting choices [15].

In other recent studies, it has been widely acknowledged that depression is a marker of increased HIV risk in both adults and adolescents [16]. Research has found depressive symptomatology among youth to be associated with earlier sexual debut, higher numbers of lifetime sexual partners, multiple and casual sexual partnerships, substance abuse, pregnancy, perceived barriers to condom use and having more risky partners [17–21]. However, most of this evidence comes from high-income countries, and these associations have not been well established in SSA where the HIV epidermic is generalized.

In SSA, the link between the HIV risk sexual behaviours and depressive symptomatology in adolescent population remains largely unexplored. A prospective cohort of young people (YP) in Eastern Cape, South Africa (SA) set out to investigate whether depressive symptomatology was associated with risky sexual behaviour [21]. Individuals with depressed symptoms were more likely to report lifetime intimate partner violence. In women, depressive symptomatology was associated with transactional sex and having dated an older partner. However, men with depressive symptoms were more likely to report ever having had transactional sex and perpetration of rape. Men were also less likely to report correct condom use at last sex.

HIV infection among adolescents with MHDs remains an important public health problem, but existing research is very scanty. In SSA, depression is believed to be higher among adolescents living with HIV (ALHIV) compared with those in the general population, with an estimated prevalence of 17–25 percent [22, 23]. Social, physical and psychological stressors associated with living with HIV are key risk factors for depression [10, 24]. For ALHIV, depression is often associated with faster disease progression, poor treatment adherence and earlier death.

In this paper we aim to determine the prevalence of depressive symptoms and the association with HIV risk behaviours in adolescents aged 15–19 years in Zambia and SA. Evaluating potential intersections between depression and HIV risk behaviours among adolescents could inform strategies that concurrently address mental wellbeing and HIV prevention within this group.

## Methods

### Study design and setting

The HPTN 071 (PopART) trial, was a three-arm community randomized trial in 12 communities in Zambia and 9 in South Africa (SA) evaluating the impact of a combination HIV prevention package, including universal HIV testing and treatment (UTT), on community-level HIV incidence [25, 26]. The PopART trial was conducted between 2013–2018 in 21 urban/peri-urban communities in Zambia and Western Cape Province, SA [25]. The 21 communities were divided into four triplets in Zambia and three in SA; communities in each triplet were randomly assigned to the three study arms: Arm A (PopART intervention with universal ART), Arm B (PopART intervention, ART per local guidelines), and Arm C (standard-of-care). Details of the PopART trial are described elsewhere [25, 27].

Nested within the PopART trial was a sub-study called PopART for Youth (P-ART-Y) whose primary outcome was knowledge of HIV status among 15–19 year-old adolescents [28]. The P-ART-Y study also presented an opportunity to explore other areas of adolescent HIV-related health which had limited data such as mental health, stigma, sex education, HIV risk behaviour and substance abuse. In order to meet these secondary objectives we conducted a cross-sectional survey. The P-ART-Y cross-sectional survey was conducted from August to November 2017 primarily to collect comparative data from the control communities on knowledge of HIV status among adolescents aged 15–19 years, for comparison with the intervention communities in which such data were collected during the third round (R3) of the PopART intervention (August 2016-December 2017) [29]. The cross-sectional survey collected additional data on mental health and formed the basis for the analysis of depression presented in this paper.

### Sampling, eligibility criteria and recruitment

The sampling frame for the survey was provided by a census of all households in the clinic catchment area of the study communities in 2013 prior to the beginning of the PopART trial. In the 7 Arm C communities, communities were subdivided into blocks, each block consisted on average of 50 (~ 40–60) households in Zambia and about 80 (~ 70–90) households in SA. Blocks were randomly selected to be part of the study. All households within a sampling block and eligible adolescents residing in these households were invited to participate in the study. To be eligible for the study, participants had to between 15–19 years old, living in a block of houses randomly selected for recruitment and able and willing to provide informed consent. We anticipated to enrol on average ~17 adolescents in each block, from ~12 blocks in each community. We aimed to enroll 200 adolescents aged 15–19 years per community, for a total of 1,400 participants.

The **study population** was a community sample of adolescents sampled from within the HPTN 071 (Pop ART) trial i.e ALHIV and those HIV-negative.

The Research Assistants (RAs) interviewed all adolescents aged 15–19 years at the time of enrollment and were living in a block of houses randomly selected for recruitment, who gave either a written informed consent (for adolescents 18 years or older) or an informed assent given by a responsible adult/parent (for adolescents less than 18 years old).

### Procedures and activities

Community sensitization, using a door to door approach was done to inform residents of the community about the survey prior to enrolment. At each house, RAs asked the responsible adult/parent for permission to enter the home and invite 15-19-year-old household members to participate in the survey. The study was explained to the guardian present and eligible adolescents. For those absent during this household visit, RAs made plans to return to the house at a time when they were expected to be at home. HIV status was self-reported.

All ALHIV who had not enrolled in care were referred to the clinic. Adolescents were also screened for TB using a TB symptoms screen (cough ≥2 weeks, drenching night sweats, unintentional weight loss). Adolescents with symptoms suggestive of TB were referred to the clinic for further management and care.

### Primary outcome measure

The primary outcome of the study was prevalence of depressive symptoms. Depressive symptoms were measured using the self-administered 13-item Short Moods and Feelings questionnaire (SMFQ) captured on a tablet (S1 Appendix), The SMFQ is designed for examining the presence of depressive symptoms in epidemiological studies; has been shown to be a strong predictor of depression; is validated in clinical and non-clinical settings and is recommended as a screening tool [30–32]. The SMFQ summaries 13 items to give a score ranging between 0–26, where greater scores represent higher depression. Many studies have used the SMFQ for exploring the nature of depression during adolescence [30, 31].

A study in New Zealand sought to validate the SMFQ among adolescents and used an optimal cut-off of ≥12 [33]. Due to lack of SMFQ validated data in SSA region, including Zambia, we adopted the same cut-off as the New Zealand study for our analysis. We therefore defined the presence of depressive symptoms if an individual’s SMFQ score was ≥12 [33]. We further grouped the scale response (0–26) into 3-equal width categories (i.e. low = 0–8, medium = 9–17 and high = 18–26) and defined the high-score category as the presence of underlying depression tendencies and the low/medium, otherwise. The second definition was then used for sensitivity analysis.

### Other measures

#### Stigma measurement

The survey presented adolescents with five statements about judgments towards PLHIV (stigmatizing attitudes towards people living with HIV (PLHIV)). We used standardized statements that had previously been used in different populations of the HPTN071 (PopART) trial [34, 35]. The statements related solely to judgmental attitudes that were held by participants: “I would be ashamed if someone in my family was living with HIV; I would not like to sit close to someone living with HIV; young people (YP) living with HIV should not share cups; YP living with HIV should not have sex; YP living with HIV should not get pregnant/have children”. (**S2 Appendix)** The stigma tool was captured electronical and offered to everybody, including ALHIV.

Adolescents were asked to respond to the statements using a 4-point Likert scale (strongly agree, agree, disagree, strongly disagree) [Cronbach’s alpha: 0.65(overall), 0.61(Zambia) and 0.71(SA)]. Strongly agree and agree were collapsed into one group (scored as 1) and strongly disagree + disagree into another (scored as 0). Those who were scored as 1 were viewed as exhibiting stigmatizing attitudes towards PLHIV.

#### Alcohol and substance use measurement

Questions on drug and alcohol use were extracted from standard questions used in other studies within the HPTN071 (PopART) study.

### Ethical considerations

In Zambia, we obtained written informed consent from adolescents aged 18–19 years and written assent for adolescents aged 15–17 years [36]. A waiver of parental consent was obtained for those aged under 18 years as the survey was considered to be low risk, only involving completion of a questionnaire. In SA, all participants (regardless of age) signed informed consent, with only verbal parental permission to enter the home required. Ethical approval was obtained from the ethics committees of the Universities of Zambia, Stellenbosch and London School of Hygiene and Tropical Medicine. Permission to conduct the study was obtained from Ministries of Health.

### Data collection

An electronic device was used to collect data. The questionnaire was administered by the RAs and self-administered for sensitive sections such as mental health, sex education/ HIV risk behavior, stigmatizing attitudes towards PLHIV, drug and alcohol use. RAs were available to guide adolescents who chose to self-administer the questionnaire.

### Data analysis

The main analyses combined Zambia and SA data. Age was the only continuous variable which was categorized into 2 categories (i.e. 15-17-year-olds and 18-19-year-olds). Frequencies (n) and percentages (%) were used to summarize categorical data and a two-sample proportion test was used for comparisons, with 95% confidence limits based on the binomial distribution. Missing data for risk factors collected across both countries was below 5% and thus a complete case analysis approach was used.

A logistic regression model was used to investigate the association between experiencing depressive symptoms and a set of potential risk factors collected during the survey. A likelihood ratio test comparing a model with clustering by block and a logistic model, showed no evidence of clustering by block, inference made by a mixture of chi-square tests, (p-value = 0.21), and therefore a logistic model was fitted.

Age, sex, country and community were selected as priori confounders and fitted as the baseline model. In a forward stepwise manner, potential risk factors were added to the baseline model and a p-value of ≤0.1 was used to identify which risk factors were associated with depressive symptoms. Variables that were conceptually on a causal pathway were not adjusted for in the analysis and variables with a p-value > 0.1 were dropped in the forward stepwise model fitting procedure. The final model in the main analysis was used as the baseline model in the consequent subgroup analysis. Subgroup analyses was carried out among adolescents who self-reported ever having had sex, separately by sex and country.

A sensitivity analysis to investigate the change in risk factors associated with depressive symptoms once a different cut-off of the scale response was used, was also carried out. Data were analysed using Stata version 15.1.

## Results

### Descriptive analysis

#### Participation

**Fig 1:**
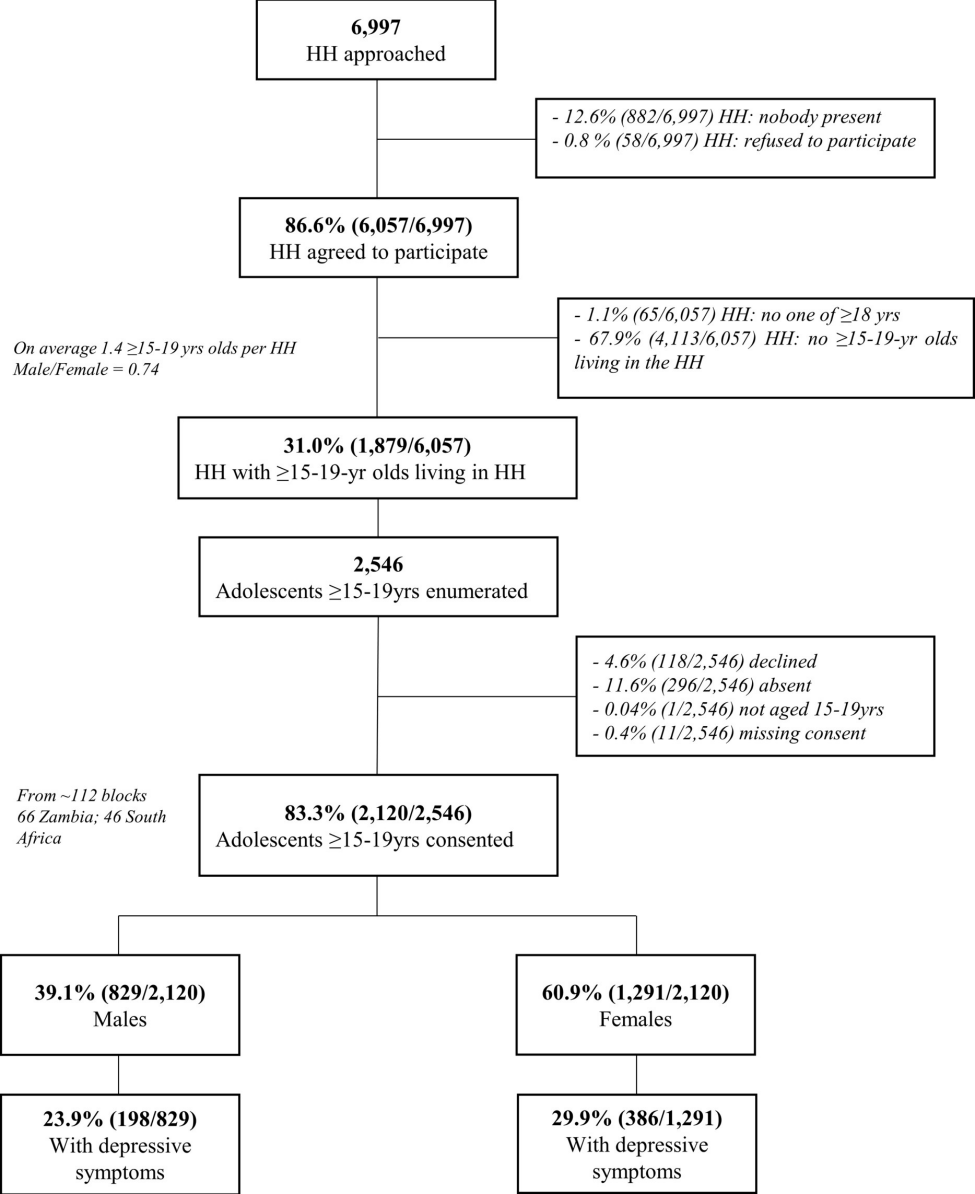
Study enumeration and participation. Note: HH = Household; Yrs = Years.

Across Zambia and SA, a total of 6997 households were approached and 6057(86.6%) consented to enumeration. Only 1879 (31%) of these households had a 15–19 year old living there at the time of the household visit. A total of 2546 adolescents aged between15-19 years were enumerated and of these 2120 (83.3%) consented to participate in the study and were administered the questionnaire (Figs 1 and S1)

#### Study population characteristics

Out of a total of 2120 adolescents who agreed to be part of the study, the majority were aged 15–17 years, 1335 (63.0%), female, 1291(60.9%), and residents in Zambia,1453 (68.5%) (Table 1). Over three-quarters, 1719 (81.1%), had not finished secondary education. A small proportion, 14 (0.7%), self-reported to be HIV positive while a majority, 1040 (49.1%), self-reported to never have tested for HIV.

**Table 1:** Distribution of the potential risk factors for depressive symptoms stratified by sex and country and combined Zambia and South Africa.

	Sex				Country				Combined Zambia and South Africa	
	Male		Female		Zambia		South Africa			
	n	%	n	%	n	%	n	%	n	%
	(N = 829)		(N = 1291)		(N = 1453)		(N = 667)		(N = 2120)	
**Socio-demographic variables**										
**Country**										
**Zambia**	559	67.4	894	69.2	-	-	-	-	**1453**	**68.5**
**South-Africa**	270	32.6	397	30.8	-	-	-	-	667	31.5
**Community**										
**Z1**	161	19.4	248	19.2	409	28.1	-	-	409	19.3
**Z2**	160	19.3	241	18.7	401	27.6	-	-	401	18.9
**Z3**	81	9.8	153	11.9	234	16.1	-	-	234	11.0
**Z4**	157	18.9	252	19.5	409	28.1	-	-	409	19.3
**SA1**	84	10.1	146	11.3	-	-	230	34.5	230	10.8
**SA2**	72	8.7	136	10.5	-	-	208	31.2	208	9.8
**SA3**	114	13.8	115	8.9	-	-	229	34.3	229	10.8
**Sex**										
**Male**	-	-	-	-	559	38.5	270	40.5	829	39.1
**Female**	-	-	-	-	894	61.5	397	59.5	**1291**	**60.9**
**Age**										
**15–17 years**	518	62.5	817	63.3	915	63.0	420	63.0	**1335**	**63.0**
**18–19 years**	311	37.5	474	36.7	538	37.0	247	37.0	785	37.0
**Education level**										
**None + Incomplete primary**	151	18.2	207	16	308	21.2	50	7.5	**358**	**16.9**
**Complete primary**	218	26.3	325	25.2	376	25.9	167	25	**543**	**25.6**
**Incomplete secondary**	329	39.7	489	37.9	513	35.3	305	45.7	**818**	**38.6**
**Complete secondary +Higher**	129	15.6	269	20.8	256	17.6	142	21.3	398	18.8
**missing**	2	**0.2**	1	0.1	0	0	3	0.4	3	0.1
**HIV-Related Risk factors**										
**HIV Test Status**										
**Never tested**	458	55.2	586	45.4	750	51.6	294	44.1	**1044**	**49.2**
**Tested>12 Months**	154	18.6	239	18.5	278	19.1	115	17.2	393	18.5
**Tested≤12 Months**	217	26.2	466	36.1	425	29.2	258	38.7	683	32.2
HIV Status										
**Never tested**	456	55.0	584	45.2	749	51.5	291	43.6	1040	49.1
**HIV negative**	366	44.1	696	53.9	693	47.7	369	55.3	1062	50.1
**HIV positive**	5	0.6	9	0.7	10	0.7	4	0.6	**14**	**0.7**
**missing**	2	0.2	2	0.2	1	0.1	3	0.4	4	0.2
**TB Status**										
**Asymptomatic**	547	66.0	906	70.2	967	66.6	486	72.9	1453	68.5
**Symptomatic**	279	33.7	380	29.4	481	33.1	178	26.7	**659**	**31.1**
**On treatment**	3	0.4	5	0.4	5	0.3	3	0.4	8	0.4
**Staying with a HIV positive adult or child**										
**no**	757	91.3	1144	88.6	1307	90.0	594	89.1	1901	89.7
**yes**	69	8.3	144	11.2	146	10.0	67	10.0	**213**	**10.0**
**missing**	3	0.4	3	0.2		0.0	6	0.9	6	0.3
**Stigmatizing attitude towards others**										
**no**	501	60.4	951	73.7	1027	70.7	425	63.7	1452	68.5
**yes**	314	37.9	322	24.9	412	28.4	224	33.6	**636**	**30.0**
**missing**	14	1.7	18	1.4	14	1.0	18	2.7	32	1.5
**Sexual risk behaviour**										
**Ever had sex**										
**no**	457	55.1	810	62.7	914	62.9	353	52.9	1267	59.8
**yes**	**370**	44.6	**480**	37.2	**539**	37.1	**311**	46.6	**850**	**40.1**
**missing**	2	0.2	1	0.1	0	0.0	3	0.4	3	0.1
Forced into sex during last sexual encounter *										
**No**	355	95.9	425	88.5	473	87.8	307	98.7	780	91.8
**Yes**	15	4.1	55	11.5	66	12.2	4	1.3	70	8.2
Age difference between last sexual partner and participant*										
**within ±5 years**	305	82.4	380	79.2	419	77.7	266	85.5	685	80.6
**>5 years older**	5	1.4	82	17.1	67	12.4	20	6.4	87	10.2
**≤5 years younger**	52	14.1	7	1.5	53	9.8	6	1.9	59	6.9
**Missing**	8	2.2	11	2.3	0	0.0	19	6.1	19	2.2
**Number of sexual partners in the last 1 year** * ^a^										
**0**	68	18.4	43	9.0	111	20.6	-	-	111	13.1
**1**	94	25.4	196	40.8	290	53.8	-	-	290	34.1
**≥2**	72	19.5	66	13.8	138	25.6	-	-	138	16.2
**Missing**	136	36.8	175	36.5	0	0.0	-	-	-	-
Condom use during last sexual intercourse*										
**Not used**	160	43.2	186	38.8	238	44.2	108	34.7	346	40.7
**Used**	210	56.8	294	61.3	301	55.8	203	65.3	504	59.3
Alcohol/drug use during last sexual encounter *										
**no**	311	84.1	444	92.5%	488	90.5	267	85.9	755	88.8
**yes**	59	15.9	36	7.5%	51	9.5	44	14.1	95	11.2
HIV Test Status*										
**Never tested**	176	47.6	107	22.3%	191	35.4	92	29.6	283	33.3
**Tested>12 Months**	70	18.9	108	22.5%	120	22.3	58	18.6	178	20.9
**Tested< = 12 Months**	124	33.5	265	55.2%	228	42.3	161	51.8	389	45.8
Circumcised**										
**no**	415	50.1	-	-	245	43.8	170	63.0	415	50.1
**Medical circumcision**	312	37.6	-	-	276	49.4	36	13.3	312	37.6
**Traditional circumcision**	50	6.0	-	-	26	4.7	24	8.9	50	6.0
**Declined to answer**	50	6.0	-	-	12	2.1	38	14.1	50	6.0
**Missing**	2	0.2	-	-	0	0.0	2	0.7	2	0.2
Currently Pregnant***										
**no**	-	-	1254	97.1	870	97.3	384	96.7	1254	97.1
**yes**	-	-	37	2.9	24	2.7	13	3.3	37	2.9

Note:

*Among those who self-reported to ever had sex

**Among males

***Among females

“-”missing information

N = denominator

Z1-Z4 = Community 1 to community 4 in Zambia; SA1-SA3 = Community 1 to community 3 in South Africa.

%(n/N) = proportion of participants at each level of the potential risk factors expressed as a percentage for the totals in Zambia, South Africa and both countries combined.

^a^ Collected in Zambia only

A total of 659 (31.1%) adolescents reported having one or more TB symptoms and 8 (0.4%) self-reported to be currently on TB treatment. Furthermore, 213 (10.0%) reported to have been staying with an HIV-positive adult or child and 636(30.0%) exhibited stigmatizing attitudes towards PLHIV. Overall, 850 (40.1%) self-reported to have ever had sex. Among those who self-reported to ever had sex, 70 (8.2%) reported having been forced into sex, and during the last sexual encounter, 346 (40.7%) reported not using a condom and 95 (11.2%) reported using alcohol or a drug. More than half of the male participants reported not to be circumcised with a majority of those circumcised having undergone medical circumcision and 37 (2.9%) of the female participants, reported to be pregnant. (Table 1).

**Fig 2:**
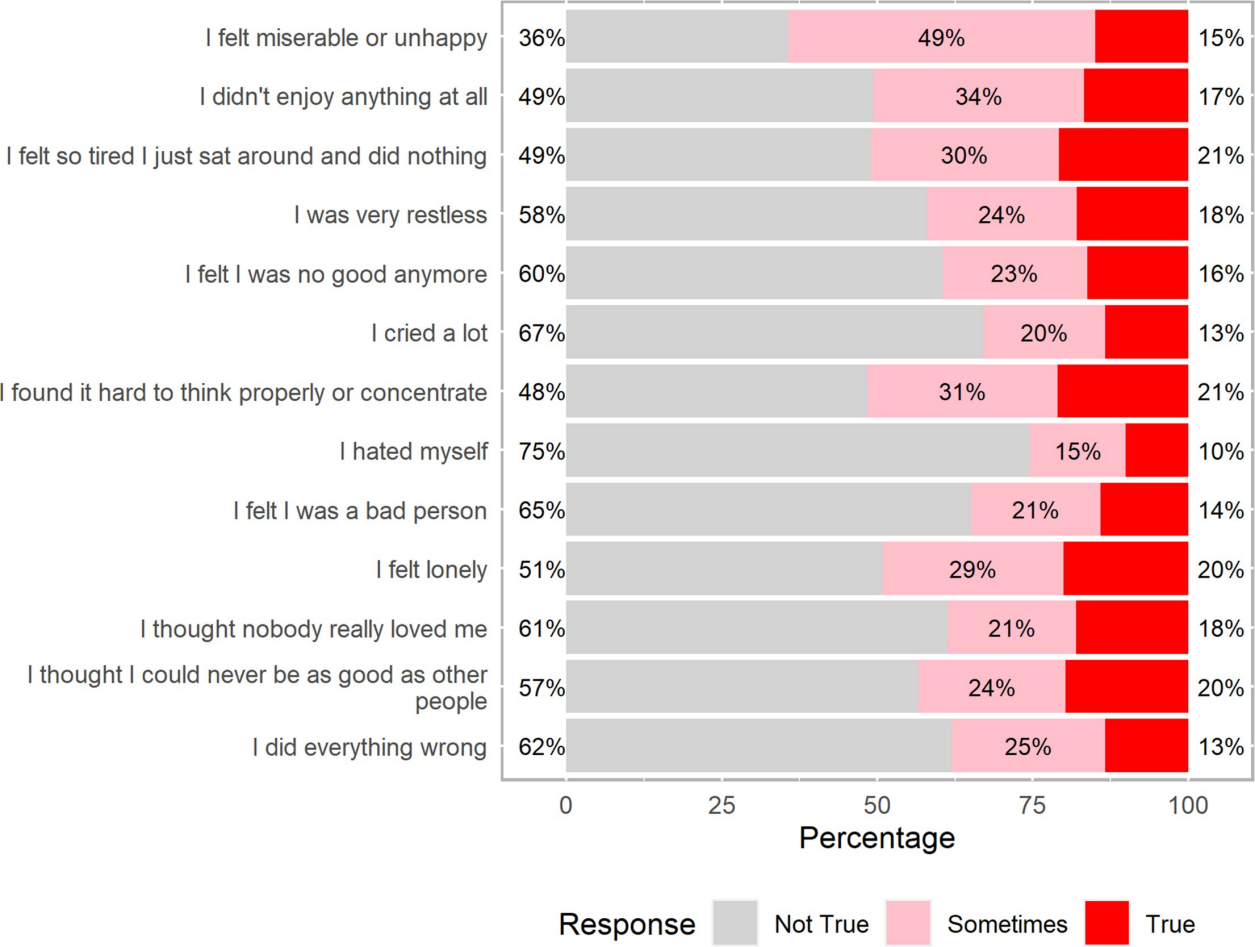
Frequency distribution of the 13 SMFQ items responses (in percentage).

**Fig 3:**
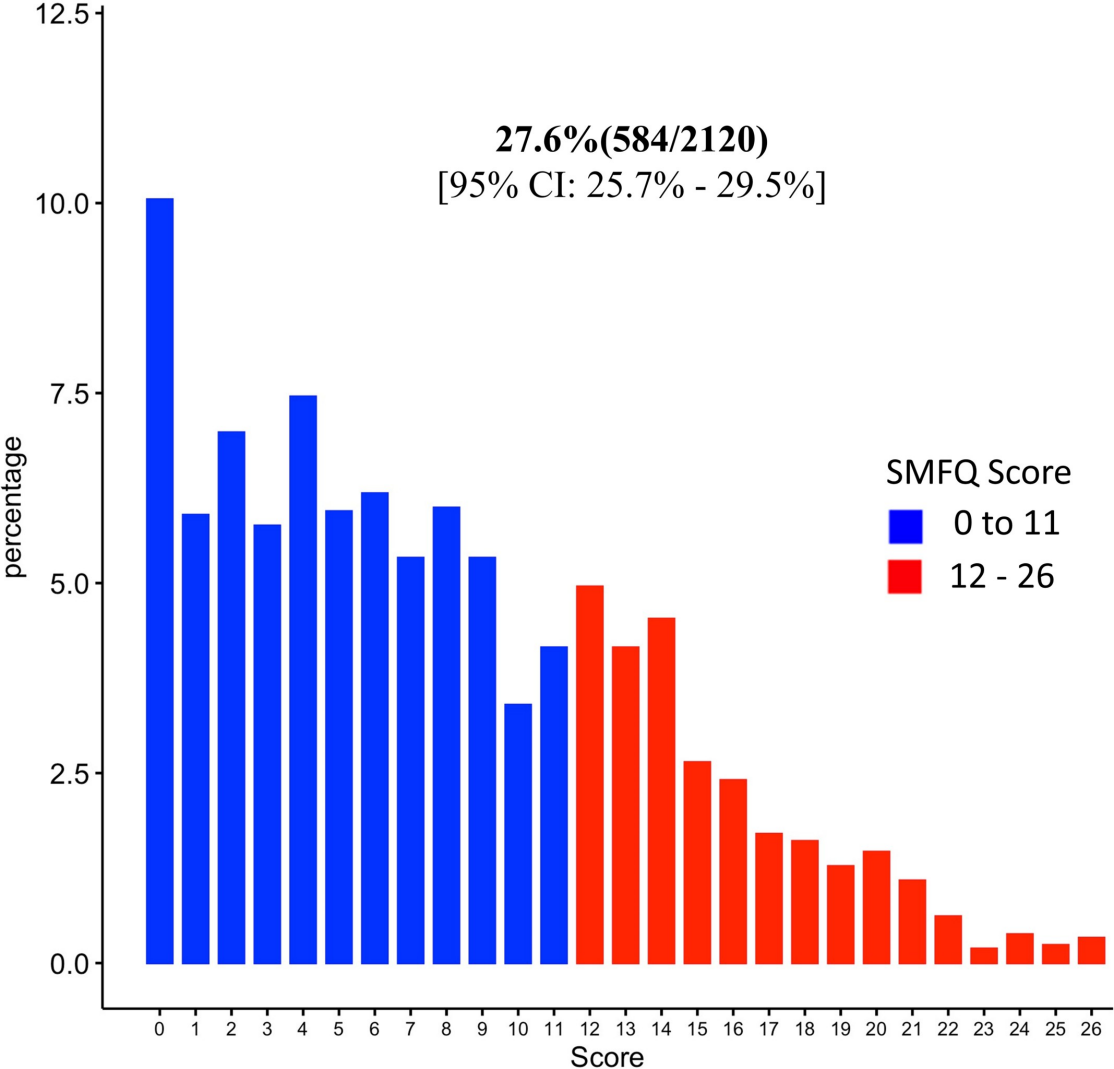
Prevalence of depressive symptoms using a ≥12 cut-off value of the SMFQ “0–26” scale response.

By combining those who answered sometimes and those who answered true to the SMFQ questions, more than 50% reported to have felt miserable or unhappy, did not enjoy anything at all, felt so tired that they just sat around and did nothing, found it hard to think properly or concentrate and 49% reported to have felt lonely (Figs 2 and S2). Using a cut-off of ≥12, the overall prevalence of depressive symptoms was 584 (27.6%) [95% Confidence-Interval [CI]: 25.7%-29.5%] (Figs 3 and S3).

The prevalence of depressive symptoms was higher among adolescents residing in Zambia 432 (29.7%); females 386 (29.9%); ALHIV 7 (50.0%); those who tested for HIV in the previous year, 216 (31.6%); those currently on TB treatment, 3 (37.5%); those who reported staying with a HIV positive adult or child, 75/213 (35.2%); those who exhibited stigmatizing attitudes towards others 192 (30.2%) and those who self-reported to have ever had sex 289(34.0%).

Among those who self-reported to have had sex before, the prevalence of depressive symptoms was higher among those who reported to have been forced into sex, 37(52.9%); those who did not used a condom during their last sexual encounter, 135 (39.0%) and those who used alcohol or drugs during their last sexual encounter 46 (48.4%). Prevalence was also high among those currently pregnant, 20 (54.1%).

#### Risk factors associated with depressive symptoms

Results from a logistic regression model adjusting for country, sex, age, TB status, staying with a HIV positive adult or child, holding stigmatizing attitude towards PLHIV, ever had sex and HIV test status, showed that; adolescents in SA were less likely to experience depressive symptoms (Adjusted Odds Ratio [AOR] = 0.63 (95% CI: 0.50, 0.79), p-value<0.0001). Female adolescents were more likely to experience depressive symptoms (AOR = 1.46 (95% CI: 1.19, 1.81), p-value<0.0001). Presumptive TB cases and those on TB treatment (AOR = 1.41(95% CI: 1.15, 1.74), p-value = 0.001) as well as adolescents who exhibited stigmatizing attitudes towards PLHIV (AOR = 1.33(95% CI: 1.07, 1.65), p-value = 0.01) were more likely to experience depressive symptoms. There was strong evidence that adolescents who reported ever having had sex were more likely to experience depressive symptoms (AOR = 1.80 (95% CI: 1.45, 2.23), p-value<0.001). Furthermore, there was of an association between depression and HIV test status (p-value = 0.02).

Country, sex, TB status, stigmatizing attitude towards others, ever had sex and HIV test status were identified as the risk factors associated with depressive symptoms (Table 2).

**Table 2:** Potential risk factors associated with depressive symptoms using the ≥12 cut-off.

	Descriptive analysis			Unadjusted model		Adjusted model 1		Adjusted model 2	
Potential risk factors	N	n	%	OR (95%CI)	P-value†	AOR (95%CI)	P-value†	AOR (95%CI)	P-value†
**Country**									
**Zambia**	1453	432	29.7	Reference	0.001	-	-	Reference	<0.001
**South-Africa**	667	152	22.8	0.70(0.56–0.86)		-		0.63(0.50–0.79)	
**Sex**									
**Male**	829	198	23.9	Reference	0.003	-	-	Reference	<0.001
**Female**	1291	386	29.9	1.36(1.11–1.66)		-		1.46(1.19–1.81)	
**Age**									
**15–17 years**	1335	365	27.3	Reference	0.8	-	-	Reference	0.3
**18–19 years**	785	219	27.9	1.03(0.84–1.25)		-		0.88(0.71–1.10)	
TB Status*									
**Asymptomatic**	1453	365	25.1	Reference	0.0003	Reference	0.0003	Reference	0.001
**On TB treatment/Symptomatic**	667	219	32.8	1.46(1.19–1.78)		1.46(1.19–1.78)		1.41(1.15–1.74)	
**Staying with a HIV positive adult or child**									
**No**	1901	509	26.8	Reference	0.009	Reference	0.013	Reference	0.1
**Yes**	213	75	35.2	1.49(1.10–2.00)		1.46(1.08–1.97)		1.31(0.95–1.80)	
**Missing**	6	0	0.0	-		-		-	
**Stigmatizing attitude towards others**									
**No**	1452	380	26.2	Reference	0.058	Reference	0.009	Reference	0.01
**Yes**	636	192	30.2	1.22(0.99–1.50)		1.32(1.07–1.63)		1.33(1.07–1.65)	
**Missing**	32	12	37.5	-		-		-	
**Ever had sex**									
**No**	1267	295	23.3	Reference	<0.0001	Reference	<0.0001	Reference	<0.001
**Yes**	850	289	34.0	1.70(1.40–2.06)		1.91(1.55–2.35)		1.80(1.45–2.23)	
**Missing**	3	0	0.0	-		-		-	
**HIV Test Status**									
**Never tested**	1044	273	26.1	Reference	0.0119	Reference	0.01	Reference	
**Tested>12 Months**	393	95	24.2	0.90(0.69–1.18)		0.90(0.68–1.18)		0.78(0.58–1.04)	
**Tested≤12 Months**	683	216	31.6	1.31(1.06–1.62)		1.32(1.06–1.65)		1.18(0.94–1.50)	0.02
**Amongst those who self-reported to ever had sex¶**									
**Forced into sex during last sexual encounter**									
**No**	780	252	32.3	Reference	0.001	Reference	0.017	Reference	0.057
**Yes**	70	37	52.9	2.35(1.44–3.85)		1.86(1.12–3.10)		1.67(0.99–2.84)	
**Condom use during last sexual intercourse**									
**Not used**	346	135	39.0	Reference	0.011	Reference	0.02	Reference	0.053
**Used**	504	154	30.6	0.69(0.52–0.92)		0.80(0.60–1.07)		0.74(0.55–1.00)	
**Alcohol/drug use during last sexual encounter**									
**No**	755	243	32.2	Reference	0.002	Reference		Reference	0.001
**Yes**	95	46	48.4	1.98(1.29–3.04)		2.35(1.50–3.67)	0.0002	2.18(1.37–3.47)	
**Amongst females¶¶**									
**Currently Pregnant**									
**No**	1254	366	29.2	Reference	0.002	Reference		Reference	0.024
**Yes**	37	20	54.1	2.85(1.48–5.51)		2.94(1.51–5.70)	0.001	2.22(1.11–4.45)	

Note:

† P-values from Likelihood ratio test

%(n/N) = proportion with depressive symptoms expressed as a percentage (Number with depressive symptoms/denominator)

“-” Information missing

OR = Odds Ratio; AOR = Adjusted Odds Ratio; CI = Confidence Interval;

* For TB status; the symptomatic and on treatment (i.e. 3/8) are collapsed into one category for the analysis at this stage

Adjusted model 1 = Adjusted for country, age and sex

Adjusted model 2 = Final model for the main analysis

Among those who reported to have sex, the baseline model is the final model in the main analysis excluding HIV test status

Among females, the baseline model is the final model in the main analysis

### Subgroup analysis

Among adolescents who self-reported ever having had sex; there was strong evidence that those who used alcohol/drugs during their last sexual encounter were more likely to experience depressive symptoms (AOR = 2.18 (95% CI: 1.37, 3.47); p-value = 0.001) (Table 2). However, there was moderate evidence for those who reported to have been forced into sex during their last sexual encounter (AOR = 1.67 (95% CI: 0.99, 2.84); p-value = 0.057). Adolescents who reported to have used a condom during their last sexual encounter were less likely to experience depressive symptoms (AOR = 0.74(95% CI: 0.55, 1.00); p-value = 0.053).

There was strong evidence that those who reported to be currently pregnant were more likely to experience depressive symptoms (AOR = 2.22 (95% CI: 1.11, 4.45); p-value = 0.02. For both countries, community, TB status, ever had sex and the use of alcohol/drug during the last sexual encounter were associated with depressive symptoms (S2 Table). Furthermore, in Zambia, sex, exhibiting stigmatizing attitudes towards others and HIV test status were identified as risk factors. S3 Table shows risk factors by sex.

### Sensitivity analysis to outcome definition

A fitted logistic regression model with this new cut off of ≥18, identified country, sex education level, TB status, ever had sex, forced into sex during their last sexual encounter and alcohol/drug use during last sexual encounter as risk factors for depressive symptoms (S4 Table).

## Discussion

Our study aimed at examining the association between adolescents’ mental health status, their HIV serostatus and the associated risk behaviours. It highlights the magnitude of depressive symptoms among adolescents in the general population and at-risk adolescents living in SSA. This study adds to literature in this area and to our knowledge by looking not only at prevalence of depression but also the associations between depression and several HIV risk factors reflecting the fact that if we want to address HIV prevention, we also need to address adolescent depression. HIV prevention programs can be more effective when they include a mental health treatment component [15]. High numbers of AYP seek mental health services, which makes them particularly accessible for HIV prevention because they are already in a mental health service system. By identifying the unique risk mechanisms associated with adolescents, customized prevention programs for youths with mental health problems can be designed. Because risk factors of specific MHDs may vary, prevention programs need to be tailored accordingly.

Adolescents in Zambia and South Africa exhibited very high depressive symptoms ranging between 25–30%, similar to other studies in SSA [9, 37, 38]. Other comparable prevalence estimates for depressive symptoms ranging from 28.8 to 32.5% were found in adolescent populations from Ghana, Nigeria, Tanzania, Uganda and Ethiopia [9]. One study conducted among adolescents (aged 15–17) in SA reported a prevalence of 2.6% (males: 3.1% vs. females: 2.0%) for depressive symptoms which was much lower than our finding [39]. Variability in prevalence across studies may be due to differences in sampled populations, study designs, sample sizes, and different screening tools used.

In Zambia, we found that adolescent girls were more likely to report depressive symptoms compared to boys while in SA results were comparable across the two groups. Previous literature has shown an association between female gender and depression across Europe and SSA [40, 41]. Depressive symptomatology has been shown to be more common in young women, while stable or decreasing prevalence has been observed in young men [42]. Adolescent girls are considered to be more at risk of mood disorders including depression; the risk being probably a result of biological, social and psychological dynamics, gender discrimination, exposure to violence and sexual abuse [43, 44]. A study in Cape Town administered a SMFQ to 1034 Grade 8 learners (mean age 14.2) and found a 41.2% prevalence of clinically significant depressive symptoms, with more females than males scoring positive [45]. However, contrary to our findings, a study in Kenya found that adolescent boys had higher chances of experiencing depressive symptoms compared to girls [46].

In this study we used the SMFQ tool to measure depressive symptoms. The SMFQ has been shown to be a strong predictor of depression in adolescents [30]. Several studies in high income countries have shown that the SMFQ is sufficient to be used as a screening tool, with gender-based cut-offs [42, 47]. Although the SMFQ tool has not yet been validated in SSA (including Zambia and SA) before, it was been validated in adolescent populations elsewhere and our reported prevalence rates were comparable to others [48, 49]. There is still lack of consensus as to which are the most valid and reliable tools to measure depressive symptoms [50]. One review surveyed 160 studies of adolescent depression and identified 33 different diagnostic and symptom measurement instruments being used by investigators globally [50].

The significant comorbidity between HIV and MHDs has been widely acknowledged [51]. The prevalence of depression symptoms among PLHIV is estimated to range between 12% and 60%, but most studies involve adult populations [41, 52]. HIV infection among adolescents with MHDs remains an important public health problem, but existing research is very scanty.

In our study, almost half of ALHIV screened positive for depressive symptoms, similar to findings elsewhere although the numbers in our study were quite small [10, 37, 53, 54]. In a systematic review looking at prevalence of depressive symptoms across 14 studies in SSA among ALHIV, the median point prevalence for depression was 22.2% (IQR 15,5–41,1) [9]. In a study conducted in Zambia among 200 ALHIV aged 15–19 years, prevalence of depressive symptoms was 25.3% [38]. In another study conducted in Choma district in Zambia in 2017, among 103 ALHIV, more than three quarters had MH problems [37]. In Uganda among 336 adolescents, 154 (~46%, [95% CI: 40.5–51.2]) had depressive symptoms; the risk was higher among those ≥ 15 years, had disclosed HIV status and had risky sexual practices [10]. The high prevalence of depression among ALHIV is probably due to the direct effect of HIV on the brain, the long-term effects of antiretroviral therapies and various biological and social stressors [7, 41, 55, 56].

Our study contributes to the overlapping burden of depressive symptoms and HIV risk behaviours among adolescents in SSA. Symptoms of depression should be considered as potential markers of increased HIV risk and this association can be causal [21]. We found that adolescents with high depressive symptomatology were more likely to report behaviours that placed them at risk for HIV infection compared to those who reported no symptoms. This finding suggests that HIV risk reduction strategies among adolescents should take into consideration the level of distress they experience. Depression may interfere with the motivation necessary for appropriate HIV risk reducing behaviours. The presence of depressive symptoms may also contribute to a higher degree of isolation and less accessibility for prevention efforts.

A study in Western Kenya assessed prevalence of HIV risk behaviours and depressive symptoms among adolescent girls and young women (AGYW) aged 15–24 years attending 4 public family planning clinics [57]. Among 487 AGYW 59 (12%) AGYW reported moderate-to-severe depression (MSD). MSD was associated with HIV risk factors including partner ≥10 years older, recent transactional sex, forced sex, intimate partner violence, and alcohol use (each p≤0.005). Thirty-four percent of AGYW with MSD had a high HIV risk score corresponding to 5 to 15 incident HIV cases per 100 person-years [57]. The findings in this study in Kenya that demonstrated that frequency of multiple HIV risk factors was higher among AGYW with depression was consistent with other studies in Uganda and SA [21, 58, 59]. Youths in Uganda who had high scores on depression were more likely to report having high numbers of sexual partners [60].

Co-morbidity of depression and substance use disorders are common among adolescents and outcomes are linked with each other. Depressed adolescents are at higher risk of developing substance use disorders especially if the onset of substance use is at an early age [61]. In our study, there was strong evidence that adolescents who reported using alcohol/drugs during their last sexual encounter were more likely to experience depressive symptoms. These findings are consistent with those observed in Zimbabwe and Uganda [10, 62].

Adolescents exposed to sexual violence in different settings are at risk of negative health outcomes, including greater likelihood of depression, substance use, suicidal ideation, anti-social behaviour, and risky sexual behaviour [63, 64]. These health consequences persist into adulthood. In a nationally representative cross-sectional study of sexual abuse of individuals aged 5–17 years in SA, 9·99% (95% CI 8·65–11·47) of boys and 14·61% (95% CI 12·83–16·56) of girls reported some lifetime sexual victimisation [39]. Adolescent ‘s own substance misuse (4·72, 3·73–5·98) and high-risk sexual behaviour (3·71, 2·99–4·61) were the behaviours most frequently associated with sexual abuse, with MHDs strongly associated with sexual victimisation (post-traumatic stress disorder 2·81, 1·65–4·78; depression 3·43, 2·26–5·19; anxiety 2·48, 1·61–3·81) [39].

The violence against children surveys were conducted in Nigeria, Uganda, and Zambia in 2014 and 2015 to examine the prevalence of coerced and forced sexual initiation (FSI) and its consequences among YP aged 13–24 years [65]. Over one in ten YP aged 13–24 years who had ever had sex experienced FSI [65]. In multivariable logistic regression, FSI was significantly associated with infrequent condom use (OR = 1.4, 95%CI = 1.1–2.1), recent experiences of sexual violence (OR = 1.6, 95%CI: 1.1–2.3), physical violence (OR = 2.2, 95%CI: 1.6–3.0), and emotional violence (OR = 2.0, 95%CI: 1.3–2.9), moderate/serious mental distress (OR = 1.5, 95%CI: 1.1–2.0), hurting oneself (OR = 2.0, 95% CI: 1.3–3.1), and thoughts of suicide (OR = 1.5, 95%CI: 1.1–2.3) [65].

We found weak evidence of an association between holding stigmatizing attitudes towards PLHIV and having depressive symptoms, similar to other studies conducted in Zambia [38, 66]. The stigma questions we asked in this study were about the *attitudes* of survey participants toward PLHIV i.e. negative attitudes that are about fear and judgment. They were *not AYP* experiences of stigma rather about what these AYP thought of others (people living with HIV). The majority of the survey participants were HIV- negative, so they would not have experienced HIV-related stigma.

Studies have shown that HIV stigma is prevalent in both Zambia and SA, be it in terms of the stigmatizing attitudes of individuals not living with HIV or as measured by stigmatizing experiences of those living with HIV [67]. For PLHIV in a highly stigmatized context such as that of our study population, the knowledge that their HIV status serves as a social blemish and leads to devaluation of their person is experienced in a variety of ways, including being the object of prejudice and discrimination, anticipation of prejudice and discrimination and internalization of negative beliefs and feelings about themselves [68] all of which are associated with higher levels of mental distress [69].

In this study, TB status was associated with depressive symptoms although evidence was quite weak. Due to the small numbers, those on TB treatment and those with at least one TB symptom were collapsed into one category for analysis. Depression is one of the most common psychiatric conditions that TB patients experience due to reduced quality of life brought about by morbidity, side effects of treatment, social stigma, fear of transmitting the disease to others, and other comorbidities associated with TB (especially HIV) [69–77]. TB may also trigger depression through an inflammatory response and or dysregulation of the hypothalamic-pituitary-adrenal axis [75].

### Strengths and limitations

Our study had a large sample size and high participation rate by adolescents in both countries. However, we acknowledge that our inclusion of risk factors was not exhaustive as the study was nested within an already ongoing large trial. The screening tool used in this study had an option to be self-administered, this was a strength as adolescents were likely to be truthful on sensitive issues, such as risk-behaviour related questions.

A major limitation of the study is that there are few culturally sensitive, standardized, and validated depression screening tools for use in adolescent populations in SSA [48, 49, 78]. However, the tool used in this study has been used in both countries [45]. Most depression tools were developed for adults and imported from high income countries [48]. Furthermore, studies have used different cut-off points for the same tool making comparisons difficult. We also cannot infer causation from our findings, having a cross-sectional study design, and therefore, for instance, it is hard to tell if a participant had depressive symptoms before or after using alcohol or drugs in their last sexual encounter.

## Conclusion

The study highlights the high prevalence of depressive symptoms among adolescents’ in Zambia and SA of approximately 25–30%. Our study shows the link between depressive symptomatology and HIV risk behaviours among adolescents. Adolescent depressive symptoms are associated with increased HIV-risk behaviour. For HIV prevention programs to be more effective they need to include a mental health treatment component. We believe that a greater understanding of the psychological factors that affect AYP is an important precursor to the design of effective HIV prevention strategies.

## Supporting information

S1 Appendix The Short Moods and Feelings questionnaire (SMFQ). (DOCX)

S2 Appendix Stigmatizing attitudes towards people living with HIV (PLHIV). (DOCX)

S1 Fig Study Participation (a) In Zambia (b) In South Africa.
(PDF)

S2 Fig Frequency distribution of the SMFQ items (in percentage). (a) Stratified by country (b) Stratified by sex.
(PDF)

S3 Fig Prevalence of depressive symptoms using a ≥12 cut-off value of the SMFQ “0–26” scale response. (a)stratified by country (b) stratified by Sex.
(PDF)

S4 Fig Frequency distribution of the 5 Stigma items responses (in percentage). (a) Overall (b) stratified by the outcome i.e. those who have depressive symptoms and those who don’t.
(PDF)

S5 Fig Frequency distribution of the 5 Stigma items responses (in percentage); Stratified by sex and age. (PDF)

S1 Table Prevalence of depressive symptoms across different levels of potential risk factors stratified by sex and country. (DOCX)

S2 Table Potential risk factors associated with depressive symptoms amongst Adolescents in Zambia and South Africa separately (using the ≥12 cut-off). (DOCX)

S3 Table Potential risk factors associated with depressive symptoms amongst males and females separately (using the ≥12 cut-off). (DOCX)

S4 Table Potential risk factors associated with depressive symptoms (using the ≥18 cut-off). (DOCX)

S1 Data Aggregate dataset. (XLSX)

S2 Data Definition of variables in aggregate dataset. (XLSX)
