# Supplementary material for: Depressive symptoms and HIV risk behaviours among adolescents enrolled in the HPTN071 (PopART) trial in Zambia and South Africa
Source: PLoS One. 2022 Dec 1;17(12):e0278291. doi: 10.1371/journal.pone.0278291 (PMC9714741; doi:10.1371/journal.pone.0278291)
Supplement: S1 Appendix — (DOCX) [file pone.0278291.s001.docx]

**S1 Appendix: The Short Moods and Feelings questionnaire (SMFQ)**

The Short Moods and Feelings questionnaire (SMFQ) is a self-administered 13-item questionnaire derived from the 33-long MFQ developed by Adrian and Elizabeth J Costello in 1987 as a screening tool for depression in children and young people in the age of 6 to 17 years (1). The SMFQ is designed for examining the presence of depressive symptoms in epidemiological studies; has been shown to be a strong predictor of depression; is validated in clinical and non-clinical settings and is recommended as a screening tool (1-3).

The SMFQ summaries 13 items to give a score ranging between 0–26, where greater scores represent higher depression. Many studies those done in SSA have used the SMFQ for exploring the nature of depression across adolescence, risk factors for greater depression, and how depression during this period can be associated with later outcomes (1, 2).

A study in New Zealand sought to validate the SMFQ among self-seeking adolescents and determined an optimal cut-off of ≥28 for the MFQ and ≥12 for the SMFQ (4). Due to lack of SMFQ validated data in SSA region, including Zambia, we adopted the same cut-off as the New Zealand study for our analysis. A ≥12 cut-off was categorized as having depressive symptoms (5). Each individual was scored by summing up the responses. The SMFQ has 3 possible responses: Not true (=0), Sometimes (=1) and True (=2). The SMFQ was also translated in the commonly spoken languages in each country.

**REFERENCES**

1. Angold A, Costello E, Messer S, Pickles A, Winder F, Silver D. The Development of a Questionnaire for Use in Epidemiological Studies of Depression in Children and Adolescents. International Journal of Methods in Psychiatric Research. 1995;5:237-49.

2. Kwong A. Examining the longitudinal nature of depressive symptoms in the Avon Longitudinal Study of Parents and Children (ALSPAC) [version 2; peer review: 3 approved]. Wellcome Open Research. 2019;4(126).

3. Hopkins K, Crosland P, Elliott N, Bewley S. Diagnosis and management of depression in children and young people: summary of updated NICE guidance. BMJ. 2015;350:h824.

4. Thabrew H, Stasiak K, Bavin L, Frampton C, Merry S. Validation of the Mood and Feelings Questionnaire (MFQ) and Short Mood and Feelings Questionnaire (SMFQ) in New Zealand help-seeking adolescents. International Journal of Methods in Psychiatric Research. 2018;27:e1610.

5. Thabrew H, McDowell H, Given K, Murrell K. Systematic Review of Screening Instruments for Psychosocial Problems in Children and Adolescents With Long-Term Physical Conditions. Global pediatric health. 2017;4:2333794X17690314-2333794X.
