# Supplementary material for: Depressive symptoms and HIV risk behaviours among adolescents enrolled in the HPTN071 (PopART) trial in Zambia and South Africa
Source: PLoS One. 2022 Dec 1;17(12):e0278291. doi: 10.1371/journal.pone.0278291 (PMC9714741; doi:10.1371/journal.pone.0278291)
Supplement: S2 Appendix — (DOCX) [file pone.0278291.s002.docx]

**S2 Appendix: Stigmatizing attitudes towards people living with HIV (PLHIV)**

A stigma questionnaire adopted included 5 statements, listed below, where the participant could either strongly disagree or disagree or agree or strongly agree (scored as 1,2,3,4 respectively) was used.

1. I would be ashamed if someone in my family had HIV.
2. I would not like to sit close to someone living with HIV, for example on public transport, at church or in a waiting room.
3. Young people (aged 15-24) living with HIV should not share cups.
4. Young people (aged 15-24) living with HIV should not have sex.
5. Young people (aged 15-24) living with HIV should not get pregnant/have children.

The frequency distribution for the responses were investigated with stratifications by the outcome, sex and age (**S6 Figure 4, S7 Figure 5).**

For the purposes of the analysis, strongly agree and agree were collapsed into one group (scored as 1) and strongly disagree + disagree into another (scored as 0). Those who were scored as 1 were viewed to be exhibiting stigmatizing attitudes.
