# Supplementary figures and images for: Depressive symptoms and HIV risk behaviours among adolescents enrolled in the HPTN071 (PopART) trial in Zambia and South Africa
Source: PLoS One. 2022 Dec 1;17(12):e0278291. doi: 10.1371/journal.pone.0278291 (PMC9714741; doi:10.1371/journal.pone.0278291)

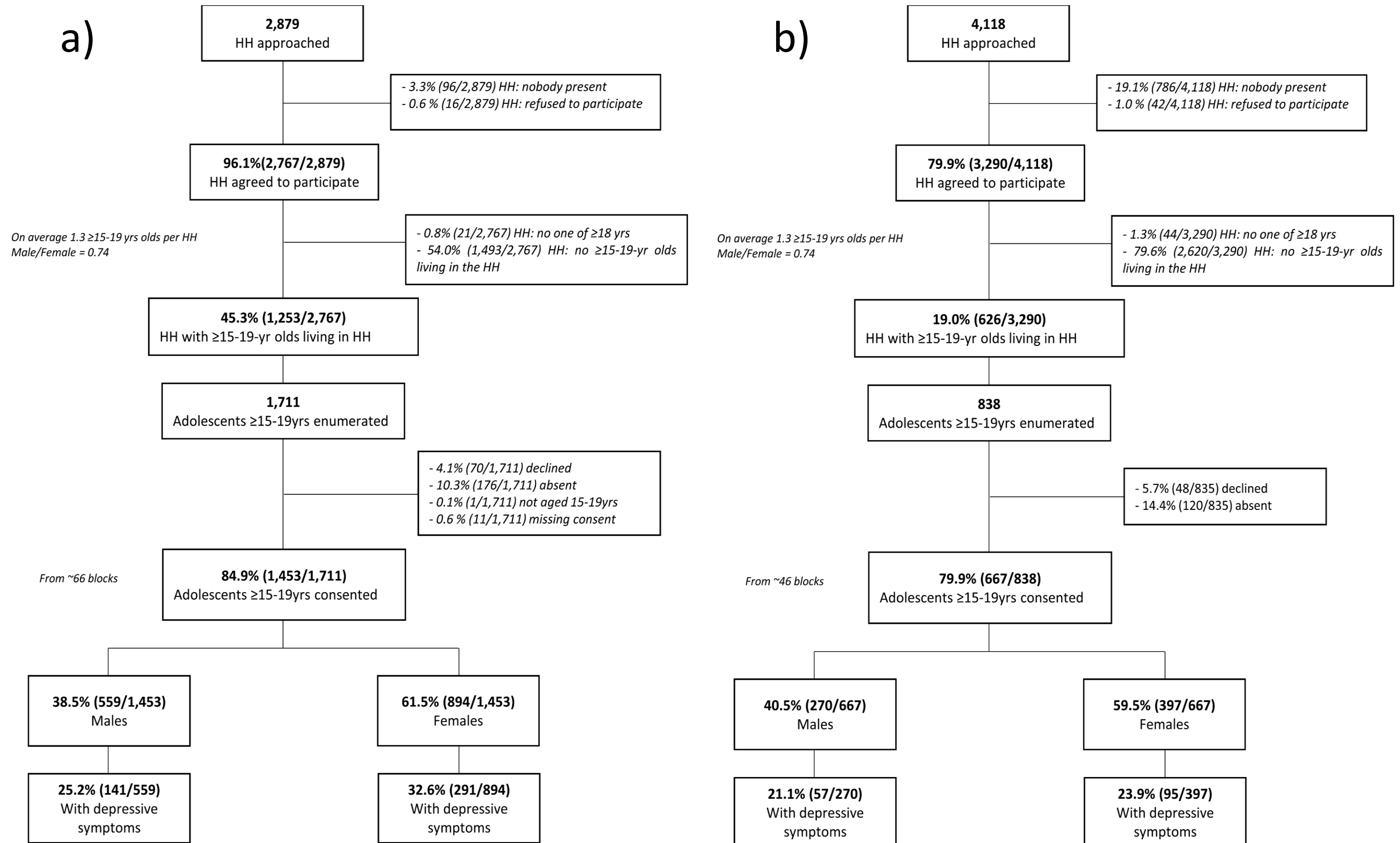

**S3 Fig 1. Study Participation (a) In Zambia (b) In South Africa**

**Note**

HH = Households

Yrs = years

Supplement: S1 Fig — Study Participation (a) In Zambia (b) In South Africa. (PDF) [file pone.0278291.s003.pdf]
