# Supplementary material for: Depressive symptoms and HIV risk behaviours among adolescents enrolled in the HPTN071 (PopART) trial in Zambia and South Africa
Source: PLoS One. 2022 Dec 1;17(12):e0278291. doi: 10.1371/journal.pone.0278291 (PMC9714741; doi:10.1371/journal.pone.0278291)
Supplement: S2 Fig — (a) Stratified by country (b) Stratified by sex. (PDF) [file pone.0278291.s004.pdf]

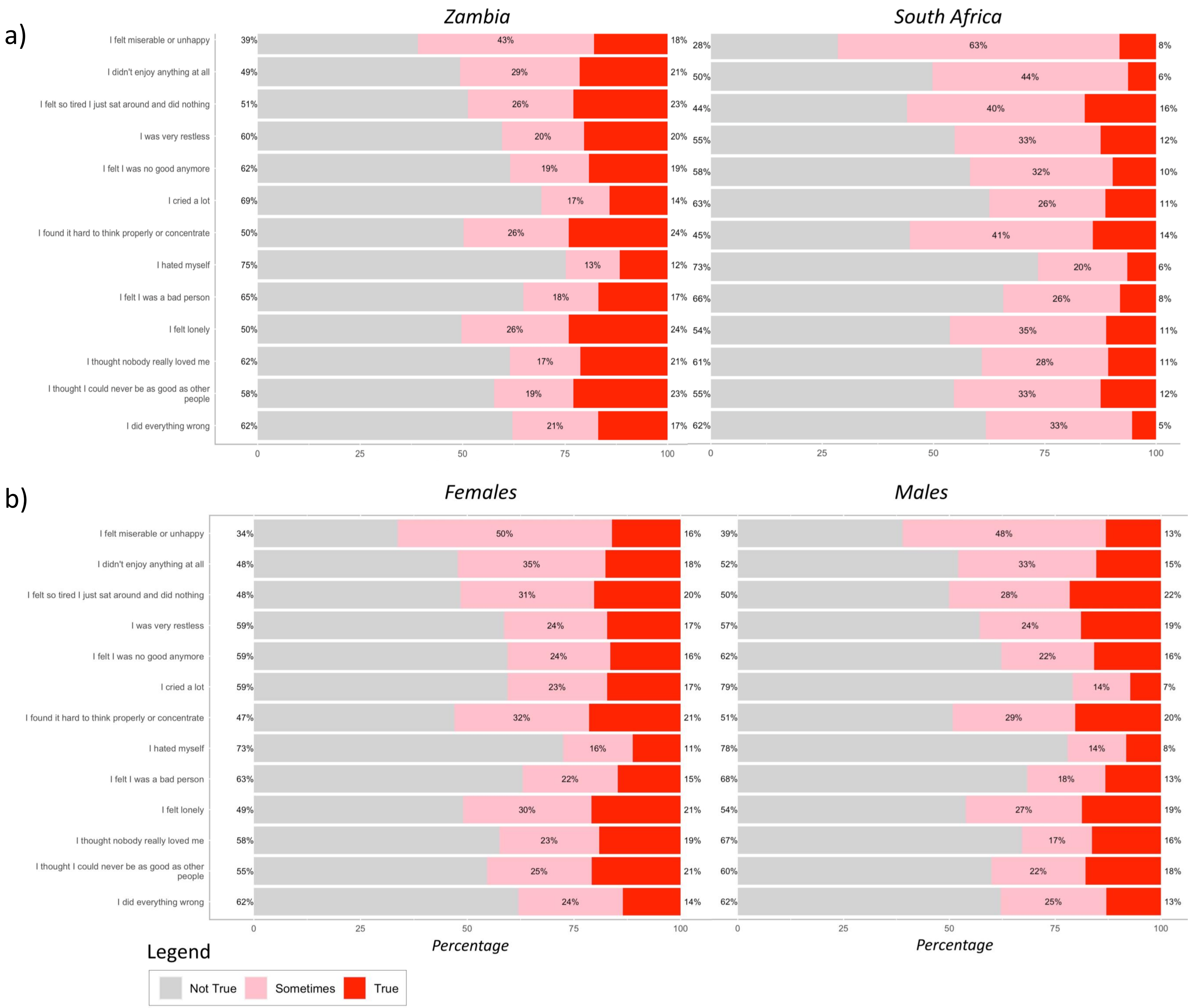

S4 Fig 2. Frequency distribution of the SMFQ items (in percentage). (a) Stratified by country (b) Stratified by sex
