# Supplementary material for: Depressive symptoms and HIV risk behaviours among adolescents enrolled in the HPTN071 (PopART) trial in Zambia and South Africa
Source: PLoS One. 2022 Dec 1;17(12):e0278291. doi: 10.1371/journal.pone.0278291 (PMC9714741; doi:10.1371/journal.pone.0278291)
Supplement: S3 Fig — (a)stratified by country (b) stratified by Sex. (PDF) [file pone.0278291.s005.pdf]

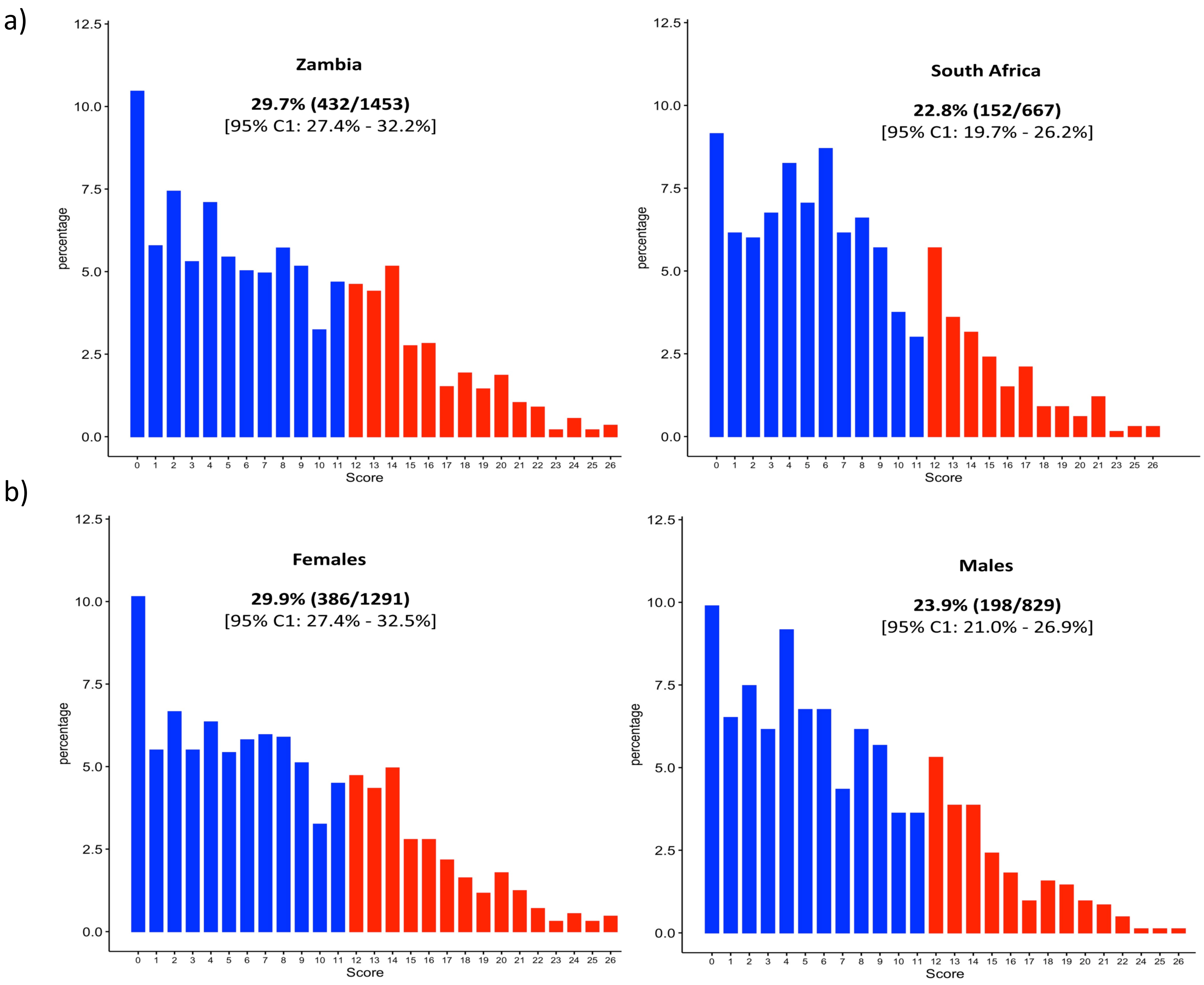

S5 Fig 3. Prevalence of depressive symptoms using a ≥12 cut-off value of the SMFQ “0 – 26” scale response. (a)stratified by country (b) stratified by Sex
