# Supplementary material for: Depressive symptoms and HIV risk behaviours among adolescents enrolled in the HPTN071 (PopART) trial in Zambia and South Africa
Source: PLoS One. 2022 Dec 1;17(12):e0278291. doi: 10.1371/journal.pone.0278291 (PMC9714741; doi:10.1371/journal.pone.0278291)
Supplement: S5 Fig — (PDF) [file pone.0278291.s007.pdf]

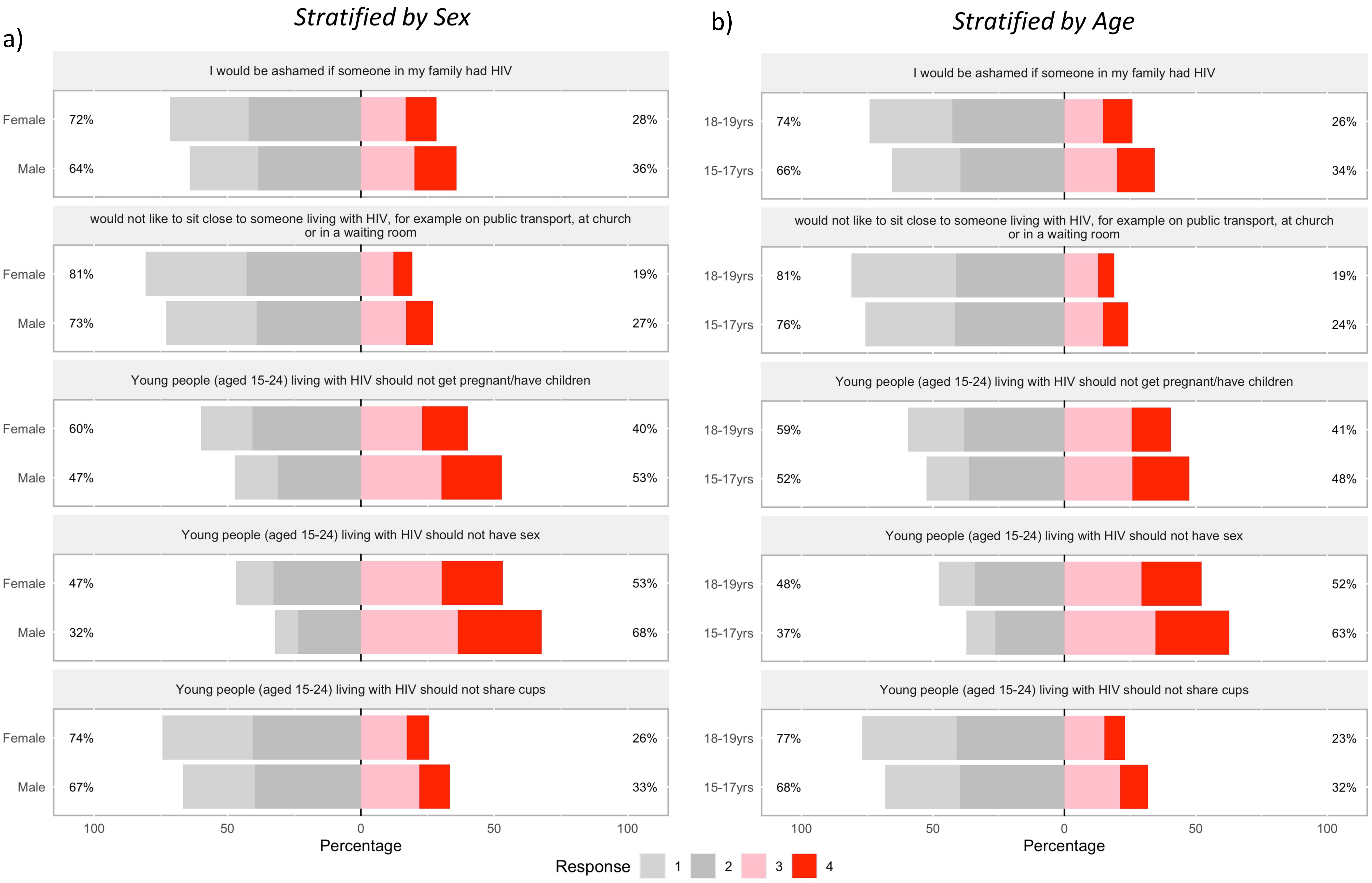

S7 Fig 5: Frequency distribution of the 5 Stigma items responses (in percentage). (a)Stratified by Sex (b) Stratified by Sex Age.
