# Supplementary material for: Depressive symptoms and HIV risk behaviours among adolescents enrolled in the HPTN071 (PopART) trial in Zambia and South Africa
Source: PLoS One. 2022 Dec 1;17(12):e0278291. doi: 10.1371/journal.pone.0278291 (PMC9714741; doi:10.1371/journal.pone.0278291)
Supplement: S1 Table — (DOCX) [file pone.0278291.s008.docx]

***S8 Table 1: Prevalence of depressive symptoms across different levels of potential risk factors stratified by sex and country***

|  | **Sex** | | | | | | **Country** | | | | | | **Combined Zambia and South Africa** | | |
| --- | --- | --- | --- | --- | --- | --- | --- | --- | --- | --- | --- | --- | --- | --- | --- |
|  | **Male** | | | **Female** | | | **Zambia** | | | **South Africa** | | |  |  |  |
| ***Potential risk factors*** | N | n | % | N | n | % | N | n | % | N | n | % | N | n | % |
| ***Country*** |  |  |  |  |  |  |  |  |  |  |  |  |  |  |  |
| *Zambia* | 559 | 141 | 25.2 | 894 | 291 | 32.6 | - | - | - | - | - | - | 1453 | 432 | 29.7 |
| *South-Africa* | 270 | 57 | 21.1 | 397 | 95 | 23.9 | - | - | - | - | - | - | 667 | 152 | 22.8 |
| ***Community*** |  |  |  |  |  |  |  |  |  |  |  |  |  |  |  |
| *Z1* | 161 | 36 | 22.4 | 248 | 72 | 29.0 | 409 | 108 | 26.4 | - | - | - | 409 | 108 | 26.4 |
| *Z2* | 160 | 34 | 21.3 | 241 | 70 | 29.0 | 401 | 104 | 25.9 | - | - | - | 401 | 104 | 25.9 |
| *Z3* | 81 | 24 | 29.6 | 153 | 63 | 41.2 | 234 | 87 | 37.2 | - | - | - | 234 | 87 | 37.2 |
| *Z4* | 157 | 47 | 29.9 | 252 | 86 | 34.1 | 409 | 133 | 32.5 | - | - | - | 409 | 133 | 32.5 |
| *SA1* | 84 | 24 | 28.6 | 146 | 38 | 26.0 | - | - | - | 230 | 62 | 27.0 | 230 | 62 | 27.0 |
| *SA2* | 72 | 20 | 27.8 | 136 | 36 | 26.5 | - | - | - | 208 | 56 | 26.9 | 208 | 56 | 26.9 |
| *SA3* | 114 | 13 | 11.4 | 115 | 21 | 18.3 | - | - | - | 229 | 34 | 14.8 | 229 | 34 | 14.8 |
| ***Sex*** |  |  |  |  |  |  |  |  |  |  |  |  |  |  |  |
| *Male* | - | - | - | - | - | - | 559 | 141 | 25.2 | 270 | 57 | 21.1 | 829 | 198 | 23.9 |
| *Female* | - | - | - | - | - | - | 894 | 291 | 32.6 | 397 | 95 | 23.9 | 1291 | 386 | 29.9 |
| ***Age*** |  |  |  |  |  |  |  |  |  |  |  |  |  |  |  |
| *15-17 years* | 518 | 123 | 23.7 | 817 | 242 | 29.6 | 915 | 271 | 29.6 | 420 | 94 | 22.4 | 1335 | 365 | 27.3 |
| *18-19 years* | 311 | 75 | 24.1 | 474 | 144 | 30.4 | 538 | 161 | 29.9 | 247 | 58 | 23.5 | 785 | 219 | 27.9 |
| ***HIV Test Status*** |  |  |  |  |  |  |  |  |  |  |  |  |  |  |  |
| *Never tested* | 458 | 109 | 23.8 | 586 | 164 | 28.0 | 750 | 215 | 28.7 | 294 | 58 | 19.7 | 1044 | 273 | 26.1 |
| *Tested>12 Months* | 154 | 32 | 20.8 | 239 | 63 | 26.4 | 278 | 72 | 25.9 | 115 | 23 | 20.0 | 393 | 95 | 24.2 |
| *Tested≤12 Months* | 217 | 57 | 26.3 | 466 | 159 | 34.1 | 425 | 145 | 34.1 | 258 | 71 | 27.5 | 683 | 216 | 31.6 |
| ***HIV Status*** |  |  |  |  |  |  |  |  |  |  |  |  |  |  |  |
| *Never tested* | 456 | 108 | 23.7 | 584 | 164 | 28.1 | 749 | 215 | 28.7 | 291 | 57 | 19.6 | 1040 | 272 | 26.2 |
| *HIV negative* | 366 | 88 | 24.0 | 696 | 216 | 31.0 | 693 | 212 | 30.6 | 369 | 92 | 24.9 | 1062 | 304 | 28.6 |
| *HIV positive* | 5 | 1 | 20.0 | 9 | 6 | 66.7 | 10 | 5 | 50.0 | 4 | 2 | 50.0 | 14 | 7 | 50.0 |
| *missing* | 2 | 1 | 50.0 | 2 | 0 | 0.0 | 1 | 0 | 0.0 | 3 | 1 | 33.3 | 4 | 1 | 25.0 |
| ***Education level*** |  |  |  |  |  |  |  |  |  |  |  |  |  |  |  |
| *None + Incomplete primary* | 151 | 51 | 33.8 | 207 | 61 | 29.5 | 308 | 100 | 32.5 | 50 | 12 | 24.0 | 358 | 112 | 31.3 |
| *Complete primary* | 218 | 40 | 18.3 | 325 | 105 | 32.3 | 376 | 108 | 28.7 | 167 | 37 | 22.2 | 543 | 145 | 26.7 |
| *Incomplete secondary* | 329 | 78 | 23.7 | 489 | 144 | 29.4 | 513 | 150 | 29.2 | 305 | 72 | 23.6 | 818 | 222 | 27.1 |
| *Complete secondary + Higher* | 129 | 29 | 22.5 | 269 | 76 | 28.3 | 256 | 74 | 28.9 | 142 | 31 | 21.8 | 398 | 105 | 26.4 |
| *missing* | 2 | - | 0.0 | 1 | - | 0.0 | - | - | - | 3 | 0 | 0.0 | 3 | 0 | 0.0 |
| ***TB Status*** |  |  |  |  |  |  |  |  |  |  |  |  |  |  |  |
| *Asymptomatic* | 547 | 119 | 21.8 | 906 | 246 | 27.2 | 967 | 271 | 28.0 | 486 | 94 | 19.3 | 1453 | 365 | 25.1 |
| *Symptomatic* | 279 | 77 | 27.6 | 380 | 139 | 36.6 | 481 | 158 | 32.8 | 178 | 58 | 32.6 | 659 | 216 | 32.8 |
| *On treatment* | 3 | 2 | 66.7 | 5 | 1 | 20.0 | 5 | 3 | 60.0 | 3 | 0 | 0.0 | 8 | 3 | 37.5 |
| ***Staying with a HIV positive adult or child*** |  |  |  |  |  |  |  |  |  |  |  |  |  |  |  |
| *no* | 757 | 175 | 23.1 | 1144 | 334 | 29.2 | 1307 | 381 | 29.2 | 594 | 128 | 21.5 | 1901 | 509 | 26.8 |
| *yes* | 69 | 23 | 33.3 | 144 | 52 | 36.1 | 146 | 51 | 34.9 | 67 | 24 | 35.8 | 213 | 75 | 35.2 |
| *missing* | 3 | - | 0.0 | 3 | - | 0.0 | - | - | - | 6 | 0 | 0.0 | 6 | 0 | 0.0 |
| ***Stigmatizing attitude towards others*** |  |  |  |  |  |  |  |  |  |  |  |  |  |  |  |
| *no* | 501 | 104 | 20.8 | 951 | 276 | 29.0 | 1027 | 284 | 27.7 | 425 | 96 | 22.6 | 1452 | 380 | 26.2 |
| *yes* | 314 | 90 | 28.7 | 322 | 102 | 31.7 | 412 | 143 | 34.7 | 224 | 49 | 21.9 | 636 | 192 | 30.2 |
| *missing* | 14 | 4 | 28.6 | 18 | 8 | 44.4 | 14 | 5 | 35.7 | 18 | 7 | 38.9 | 32 | 12 | 37.5 |
| ***Ever had sex*** |  |  |  |  |  |  |  |  |  |  |  |  |  |  |  |
| *no* | 457 | 89 | 19.5 | 810 | 206 | 25.4 | 914 | 225 | 24.6 | 353 | 70 | 19.8 | 1267 | 295 | 23.3 |
| *yes* | 370 | 109 | 29.5 | 480 | 180 | 37.5 | 539 | 207 | 38.4 | 311 | 82 | 26.4 | 850 | 289 | 34.0 |
| *missing* | 2 |  | 0.0 | 1 |  | 0.0 |  |  | - | 3 | 0 | 0.0 | 3 | 0 | 0.0 |
| ***HIV Test Status**** |  |  |  |  |  |  |  |  |  |  |  |  |  |  |  |
| *Never tested* | 176 | 55 | 31.3 | 107 | 45 | 42.1 | 191 | 75 | 39.3 | 92 | 25 | 27.2 | 283 | 100 | 35.3 |
| *Tested>12 Months* | 70 | 17 | 24.3 | 108 | 38 | 35.2 | 120 | 42 | 35.0 | 58 | 13 | 22.4 | 178 | 55 | 30.9 |
| ***Tested≤12 Months*** | 124 | 37 | 29.8 | 265 | 97 | 36.6 | 228 | 90 | 39.5 | 161 | 44 | 27.3 | 389 | 134 | 34.5 |
| ***Forced into sex during last sexual encounter**** |  |  |  |  |  |  |  |  |  |  |  |  |  |  |  |
| ***no*** | 355 | 102 | 28.7 | 425 | 150 | 35.3 | 473 | 173 | 36.6 | 307 | 79 | 25.7 | 780 | 252 | 32.3 |
| ***yes*** | 15 | 7 | 46.7 | 55 | 30 | 54.5 | 66 | 34 | 51.5 | 4 | 3 | 75.0 | 70 | 37 | 52.9 |
| ***Age difference between last sexual partner and participant**** |  |  |  |  |  |  |  |  |  |  |  |  |  |  |  |
| *within ±5 years* | 305 | 87 | 28.5 | 380 | 137 | 36.1 | 419 | 162 | 38.7 | 266 | 62 | 23.3 | 685 | 224 | 32.7 |
| *≥5 years older* | 5 | 3 | 60.0 | 82 | 34 | 41.5 | 67 | 27 | 40.3 | 20 | 10 | 50.0 | 87 | 37 | 42.5 |
| *≤5 years younger* | 52 | 16 | 30.8 | 7 | 4 | 57.1 | 53 | 18 | 34.0 | 6 | 2 | 33.3 | 59 | 20 | 33.9 |
| *missing* | 8 | 3 | 37.5 | 11 | 5 | 45.5 | - | - | - | 19 | 8 | 42.1 | 19 | 8 | 42.1 |
| ***Number of sexual partners in the last 1 year**** |  |  |  |  |  |  |  |  |  |  |  |  |  |  |  |
| *0* | 68 | 23 | 33.8 | 43 | 16 | 37.2 | 111 | 39 | 35.1 | - | - | - | 111 | 39 | 35.1 |
| *1* | 94 | 26 | 27.7 | 196 | 83 | 42.3 | 290 | 109 | 37.6 | - | - | - | 290 | 109 | 37.6 |
| *≥2* | 72 | 23 | 31.9 | 66 | 36 | 54.5 | 138 | 59 | 42.8 | - | - | - | 138 | 59 | 42.8 |
| *missing* | 136 | 37 | 27.2 | 175 | 45 | 25.7 | - | - | - | - | - | - | 311 | 82 | 26.4 |
| ***Condom use during last sexual intercourse**** |  |  |  |  |  |  |  |  |  |  |  |  |  |  |  |
| *Not used* | 160 | 56 | 35.0 | 186 | 79 | 42.5 | 238 | 93 | 39.1 | 108 | 42 | 38.9 | 346 | 135 | 39.0 |
| *used* | 210 | 53 | 25.2 | 294 | 101 | 34.4 | 301 | 114 | 37.9 | 203 | 40 | 19.7 | 504 | 154 | 30.6 |
| ***Alcohol/drug use during last sexual encounter **** |  |  |  |  |  |  |  |  |  |  |  |  |  |  |  |
| *no* | 311 | 84 | 27.0 | 444 | 159 | 35.8 | 488 | 181 | 37.1 | 267 | 62 | 23.2 | 755 | 243 | 32.2 |
| *yes* | 59 | 25 | 42.4 | 36 | 21 | 58.3 | 51 | 26 | 51.0 | 44 | 20 | 45.5 | 95 | 46 | 48.4 |
| ***Circumcised***** |  |  |  |  |  |  |  |  |  |  |  |  |  |  |  |
| *no* | 415 | 97 | 23.4 | - | - | - | 245 | 66 | 26.9 | 170 | 31 | 18.2 | 415 | 97 | 23.4 |
| *Medical circumcision* | 312 | 70 | 22.4 | - | - | - | 276 | 63 | 22.8 | 36 | 7 | 19.4 | 312 | 70 | 22.4 |
| *Traditional circumcision* | 50 | 16 | 32.0 | - | - | - | 26 | 8 | 30.8 | 24 | 8 | 33.3 | 50 | 16 | 32.0 |
| ***Declined to answer*** | **50** | 15 | 30.0 | - | - | - | 12 | 4 | 33.3 | 38 | 11 | 28.9 | 50 | 15 | 30.0 |
| ***Missing*** | **2** |  | 0.0 | - | - | - | - | - | - | 2 | 0 | 0.0 | 2 | 0 | 0.0 |
| ***Currently Pregnant****** |  |  |  |  |  |  |  |  |  |  |  |  |  |  |  |
| ***no*** | **-** | - | - | 1254 | 366 | 29.2 | 870 | 277 | 31.8 | 384 | 89 | 23.2 | 1254 | 366 | 29.2 |
| ***yes*** | **-** | - | - | 37 | 20 | 54.1 | 24 | 14 | 58.3 | 13 | 6 | 46.2 | 37 | 20 | 54.1 |

**Note:**

*Among those who self-reported to ever had sex

**Among males

***Among females

“-“ missing information

Z1-Z4 = Community 1 to community 4 in Zambia; SA1-SA3 = Community 1 to community 3 in South Africa.

%(n/N) = proportion with depressive symptoms expressed as a percentage (Number with depressive symptoms/denominator)
