# Supplementary material for: Depressive symptoms and HIV risk behaviours among adolescents enrolled in the HPTN071 (PopART) trial in Zambia and South Africa
Source: PLoS One. 2022 Dec 1;17(12):e0278291. doi: 10.1371/journal.pone.0278291 (PMC9714741; doi:10.1371/journal.pone.0278291)
Supplement: S2 Table — (DOCX) [file pone.0278291.s009.docx]

***S9 Table 2: Potential risk factors associated with depressive symptoms amongst Adolescents in Zambia and South Africa separately (using the ≥12 cut-off)***

|  | ***Descriptive analysis*** | | ***Adjusted model 2*** | | | |
| --- | --- | --- | --- | --- | --- | --- |
|  | ***Zambia*** | ***South Africa*** | ***Zambia*** | | ***South Africa*** | |
| ***Potential risk factor*** | ***%(n/N)*** | ***%(n/N)*** | ***AOR (95%CI)*** | ***P-value†*** | ***AOR (95%CI)*** | ***P-value*** |
| ***Community*** |  |  |  |  |  |  |
| ***3*** | 26.4% (108/409) | - | Reference | 0.02 |  |  |
| ***4*** | 25.9% (104/401) | - | 1.02(0.74-1.41) |  |  |  |
| ***7*** | 37.2% (87/234) | - | 1.64(1.15-2.35) |  |  |  |
| ***12*** | 32.5% (133/409) | - | 1.29(0.94-1.78) |  |  |  |
| ***15*** | - | 27.0% (62/230) | - |  | Reference | 0.005 |
| ***17*** | - | 26.9% (56/208) | - |  | 0.88(0.56-1.38) |  |
| ***21*** | - | 14.8% (34/229) | - |  | 0.44(0.26-0.75) |  |
| ***Sex*** |  |  |  |  |  |  |
| ***Male*** | 25.2% (141/559) | 21.1% (57/270) | Reference | 0.001 | Reference | 0.40 |
| ***Female*** | 32.6% (291/894) | 23.9% (95/397) | 1.55(1.21-1.99) |  | 1.19(0.79-1.80) |  |
| ***Age*** |  |  |  |  |  |  |
| ***15-17yrs*** | 29.6% (271/915) | 22.4% (94/420) | Reference | 0.26 | Reference | 0.96 |
| ***18-19yrs*** | 29.9% (161/538) | 23.5% (58/247) | 0.86(0.66-1.12) |  | 0.99(0.65-1.50) |  |
| ***TB Status**** |  |  |  |  |  |  |
| ***Asymptomatic*** | 28.0% (271/967) | 19.3% (94/486) | Reference | 0.038 | Reference | 0.002 |
| ***On TB treatment/Symptomatic*** | 32.8% (161/486) | 32.6% (58/181) | 1.30(1.01-1.67) |  | 1.92(1.26-2.92) |  |
| ***Staying with a HIV positive adult or child*** |  |  |  |  |  |  |
| ***no*** | 29.2% (381/1307) | 21.5% (128/594) | Reference | 0.31 | Reference | 0.20 |
| ***yes*** | 34.9% (51/146) | 35.8% (24/67) | 1.22(0.83-1.80) |  | 1.48(0.81-2.69) |  |
| ***missing*** | - | 0% (0/6) | - |  | - |  |
| **Stigmatizing attitude towards others** |  |  |  |  |  |  |
| ***no*** | 27.7%(284/1027) | 22.6% (96/425) | Reference | 0.008 | Reference | 0.47 |
| ***yes*** | 34.7% (143/412) | 21.9% (49/224) | 1.42(1.09-1.84) |  | 1.17(0.77-1.79) |  |
| ***missing*** | 35.7% (5/14) | 38.9% (7/18) | - |  | - |  |
| ***Ever had sex*** |  |  |  |  |  |  |
| ***no*** | 24.6% (225/914) | 19.8% (70/353) | Reference | <0.001 | Reference | 0.19 |
| ***yes*** | 38.4% (207/539) | 26.4% (82/311) | 2.07(1.6-2.68) |  | 1.31(0.88-1.97) |  |
| ***missing*** | - | 0% (0/3) | - |  | - |  |
| ***HIV Test Status*** |  |  |  |  |  |  |
| ***Never tested*** | 28.7% (215/750) | 19.7% (58/294) | Reference | 0.3 | Reference | 0.1 |
| ***Tested>12M*** | 25.9% (72/278) | 20.0% (23/115) | 0.82(0.58-1.14) |  | 0.6(0.32-1.14) |  |
| ***Tested≤12M*** | 34.1% (145/425) | 27.5% (71/258) | 1.09(0.82-1.46) |  | 1.13(0.71-1.81) |  |
| ***Amongst those who self-reported to ever had sex*** | | | | | | |
| ***Forced into sex during last sexual encounter*** |  |  |  |  |  |  |
| ***no*** | 36.6% (173/473) | 25.7% (79/307) | Reference | 0.086 | Reference | 0.43 |
| ***yes*** | 51.5% (34/66) | 75.0% (3/4) | 1.61(0.94-2.78) |  | 2.81(0.21-36.86) |  |
| ***Condom use during last sexual intercourse*** |  |  |  |  |  |  |
| ***Not used*** | 39.1% (93/238) | 38.9% (42/108) | Reference | 0.66 | Reference | 0.002 |
| ***used*** | 37.9% (114/301) | 19.7% (40/203) | 0.92(0.64-1.33) |  | 0.41(0.24-0.72) |  |
| ***Alcohol/drug use during last sexual encounter*** |  |  |  |  |  |  |
| ***no*** | 37.1% (181/488) | 23.2% (62/267) | Reference | 0.03 | Reference | 0.036 |
| ***yes*** | 51.0% (26/51) | 45.5% (20/44) | 1.97(1.07-3.61) |  | 2.32(1.05-5.09) |  |
| ***Amongst females*** | | | | | | |
| ***Currently Pregnant*** |  |  |  |  |  |  |
| ***no*** | 31.8% (277/870) | 23.2% (89/384) | Reference | 0.16 | Reference | 0.07 |
| ***yes*** | 58.3% (14/24) | 46.2% (6/13) | 1.88(0.79-4.46) |  | 2.90(0.91-9.30) |  |

**Note:**

**†** P-values from Likelihood ratio test

%(n/N) = proportion with depressive symptoms expressed as a percentage (Number with depressive symptoms/denominator)

“-” Information missing

OR = Odds Ratio; AOR = Adjusted Odds Ratio; CI = Confidence Interval;

* For *TB status*; the symptomatic and on treatment were collapsed into one category for the analysis at this stage

Adjusted model 2 = Final model for the main analysis as described on the methods section in the main text
