# Supplementary material for: Depressive symptoms and HIV risk behaviours among adolescents enrolled in the HPTN071 (PopART) trial in Zambia and South Africa
Source: PLoS One. 2022 Dec 1;17(12):e0278291. doi: 10.1371/journal.pone.0278291 (PMC9714741; doi:10.1371/journal.pone.0278291)
Supplement: S3 Table — (DOCX) [file pone.0278291.s010.docx]

***S10 Table 3: Potential risk factors associated with depressive symptoms amongst males and females separately (using the ≥12 cut-off)***

|  | ***Descriptive analysis*** | | ***Adjusted model 2*** | | | |
| --- | --- | --- | --- | --- | --- | --- |
|  | **Male** | **Female** | **Male** | | **Female** | |
| ***Potential risk factor*** | **%(n/N)** | **%(n/N)** | **AOR (95%CI)** | **P-value** | **AOR (95%CI)** | **P-value** |
| ***Country*** |  |  |  |  |  |  |
| ***Zambia*** | 25.2% (141/559) | 32.6% (291/894) | Reference | 0.04 | Reference | <0.001 |
| ***South-Africa*** | 21.1% (57/270) | 23.9% (95/397) | 0.68(0.47-0.98) |  | 0.60(0.45-0.79) |  |
| ***Age*** |  |  |  |  |  |  |
| ***15-17yrs*** | 23.7% (123/518) | 29.6% (242/817) | Reference | 0.81 | Reference | 0.18 |
| ***18-19yrs*** | 24.1% (75/311) | 30.4% (144/474) | 0.96(0.67-1.36) |  | 0.83(0.62-1.09) |  |
| ***TB Status*** |  |  |  |  |  |  |
| ***Asymptomatic*** | 21.8% (119/547) | 27.2% (246/906) | Reference | 0.093 | Reference | 0.005 |
| ***On TB treatment/Symptomatic*** | 28.0% (79/282) | 36.4% (140/385) | 1.34(0.95-1.88) |  | 1.46(1.12-1.91) |  |
| ***Staying with a HIV positive adult or child*** |  |  |  |  |  |  |
| ***no*** | 23.1% (175/757) | 29.2% (334/1144) | Reference | 0.19 | Reference | 0.28 |
| ***yes*** | 33.3% (23/69) | 36.1% (52/144) | 1.48(0.83-2.64) |  | 1.24(0.84-1.83) |  |
| ***missing*** | 0% (0/3) | 0% (0/3) | - |  | - |  |
| **Stigmatizing attitude towards others** |  |  |  |  |  |  |
| ***no*** | 20.8% (104/501) | 29.0% (276/951) | Reference | 0.006 | Reference | 0.40 |
| ***yes*** | 28.7% (90/314) | 31.7% (102/322) | 1.61(1.15-2.25) |  | 1.13(0.85-1.50) |  |
| ***missing*** | 28.6% (4/14) | 44.4% (8/18) | - |  | - |  |
| ***Ever had sex*** |  |  |  |  |  |  |
| ***no*** | 19.5% (89/457) | 25.4% (206/810) | Reference | 0.001 | Reference | <0.001 |
| ***yes*** | 29.5% (109/370) | 37.5% (180/480) | 1.76(1.25-2.46) |  | 1.79(1.34-2.37) |  |
| ***missing*** | 0% (/2) | 0% (/1) | - |  | - |  |
| ***HIV Test Status*** |  |  |  |  |  |  |
| ***Never tested*** | 23.8% (109/458) | 28.0% (164/586) | Reference | 0.60 | Reference | 0.03 |
| ***Tested>12M*** | 20.8% (32/154) | 26.4% (63/239) | 0.82(0.51-1.32) |  | 0.76(0.52-1.10) |  |
| ***Tested≤12M*** | 26.3% (57/217) | 34.1% (159/466) | 1.08(0.73-1.59) |  | 1.23(0.92-1.66) |  |
| ***Amongst those who self-reported to ever had sex*** | | | | | | |
| ***Forced into sex during last sexual encounter*** |  |  |  |  |  |  |
| ***no*** | 28.7% (102/355) | 35.3% (150/425) | Reference | 0.41 | Reference | 0.12 |
| ***yes*** | 46.7% (7/15) | 54.5% (30/55) | 1.61(0.52-4.93) |  | 1.63(0.89-3.00) |  |
| ***Condom use during last sexual intercourse*** |  |  |  |  |  |  |
| ***Not used*** | 35.0% (56/160) | 42.5% (79/186) | Reference | 0.099 | Reference | 0.21 |
| ***used*** | 25.2% (53/210) | 34.4% (101/294) | 0.67(0.42-1.08) |  | 0.78(0.52-1.16) |  |
| ***Alcohol/drug use during last sexual encounter*** |  |  |  |  |  |  |
| ***no*** | 27.0% (84/311) | 35.8% (159/444) | Reference | 0.039 | Reference | 0.013 |
| ***yes*** | 42.4% (25/59) | 58.3% (21/36) | 1.90(1.03-3.49) |  | 2.57(1.22-5.41) |  |
| ***Amongst females*** | | | | | | |
| ***Currently Pregnant*** |  |  |  |  |  |  |
| ***no*** | - | 29.2% (366/1254) | - | - | Reference | 0.016 |
| ***yes*** | - | 54.1% (20/37) | - |  | 2.36(1.18-4.73) |  |

**Note:**

**†** P-values from Likelihood ratio test

%(n/N) = proportion with depressive symptoms expressed as a percentage (Number with depressive symptoms/denominator)

“-” Information missing

OR = Odds Ratio; AOR = Adjusted Odds Ratio; CI = Confidence Interval;

* For *TB status*; the symptomatic and on treatment were collapsed into one category for the analysis at this stage

Adjusted model 2 = Final model for the main analysis as described on the methods section in the main text.
