# Supplementary material for: Depressive symptoms and HIV risk behaviours among adolescents enrolled in the HPTN071 (PopART) trial in Zambia and South Africa
Source: PLoS One. 2022 Dec 1;17(12):e0278291. doi: 10.1371/journal.pone.0278291 (PMC9714741; doi:10.1371/journal.pone.0278291)
Supplement: S4 Table — (DOCX) [file pone.0278291.s011.docx]

***S11 Table 4: Potential risk factors associated with depressive symptoms (using the ≥18 cut-off)***

|  | ***Descriptive analysis*** | ***Unadjusted model*** | | ***Adjusted model 1*** | | ***Adjusted model 2*** | |
| --- | --- | --- | --- | --- | --- | --- | --- |
| ***Potential risk factor*** | ***%(n/N)*** | ***OR (95%CI)*** | ***P-value†*** | ***OR (95%CI)*** | ***P-value†*** | ***OR (95%CI)*** | ***P-value†*** |
| ***Country*** |  |  |  |  |  |  |  |
| ***Zambia*** | 8.5% (123/1453) | Reference | <0.001 | - | - | Reference | <0.001 |
| ***South-Africa*** | 4.5% (29/667) | 0.49(0.32-0.74) |  | - |  | 0.47(0.31-0.72) |  |
| ***Sex*** |  |  |  |  |  |  |  |
| ***Male*** | 5.7% (47/829) | Reference | 0.033 | - | - | Reference | 0.008 |
| ***Female*** | 8.1% (105/1291) | 1.47(1.03-2.10) |  | - |  | 1.63(1.13-2.35) |  |
| ***Age*** |  |  |  |  |  |  |  |
| ***15-17yrs*** | 7.2% (96/1335) | Reference | 0.96 | - | - | Reference | 0.08 |
| ***18-19yrs*** | 7.1% (56/785) | 0.99(0.70-1.40) |  | - |  | 0.71(0.48-1.04) |  |
| ***Education level*** |  |  |  |  |  |  |  |
| ***None + Incomplete primary*** | 9.8% (35/358) | Reference | 0.021 | Reference | 0.033 | Reference | 0.019 |
| ***Complete primary*** | 4.6% (25/543) | 0.45(0.26-0.76) |  | 0.48(0.28-0.83) |  | 0.45(0.26-0.78) |  |
| ***Incomplete secondary*** | 7.5% (61/818) | 0.74(0.48-1.15) |  | 0.85(0.55-1.33) |  | 0.82(0.52-1.28) |  |
| ***Complete secondary + Higher*** | 7.8% (31/398) | 0.78(0.47-1.29) |  | 0.88(0.51-1.51) |  | 0.87(0.50-1.51) |  |
| ***missing*** | 0% (0/3) | - |  | - |  | - |  |
| ***TB Status**** |  |  |  |  |  |  |  |
| ***Asymptomatic*** | 5.8% (85/1453) | Reference | 0.001 | Reference | 0.001 | Reference | <0.001 |
| ***On TB treatment/Symptomatic*** | 10.0% (67/667) | 1.80(1.29-2.51) |  | 1.77(1.27-2.49) |  | 1.75(1.24-2.46) |  |
| ***Staying with a HIV positive adult or child*** |  |  |  |  |  |  |  |
| ***no*** | 6.9% (131/1901) | Reference | 0.11 | Reference | 0.14 | - | - |
| ***yes*** | 9.9% (21/213) | 1.48(0.91-2.40) |  | 1.44(0.89-2.35) |  | - |  |
| ***missing*** | 0% (0/6) | - |  | - |  | - |  |
| **Stigmatizing attitude towards others** |  |  |  |  |  |  |  |
| ***no*** | 7.4% (108/1452) | Reference | 0.42 | Reference | 0.72 | - | - |
| ***yes*** | 6.4% (41/636) | 0.86(0.59-1.24) |  | 0.93(0.64-1.36) |  | - |  |
| ***missing*** | 9.4% (3/32) | - |  | - |  | - |  |
| ***Ever had sex*** |  |  |  |  |  |  |  |
| ***no*** | 5.1% (65/1267) | Reference | <0.0001 | Reference | <0.0001 | Reference | <0.001 |
| ***yes*** | 10.2% (87/850) | 2.11(1.51-2.94) |  | 2.56(1.79-3.66) |  | 2.58(1.80-3.71) |  |
| ***missing*** | 0% (0/3) | - |  | - |  | - |  |
| ***Test Status*** |  |  |  |  |  |  |  |
| ***Never tested*** | 6.7% (70/1044) | Reference | 0.7 | Reference | 0.6 | - | - |
| ***Tested>12M*** | 7.4% (29/393) | 1.11(0.71-1.74) |  | 1.12(0.71-1.76) |  | - |  |
| ***Tested<=12M*** | 7.8% (53/683) | 1.17(0.81-1.70) |  | 1.22(0.83-1.79) |  | - |  |
| ***Amongst those who self-reported to ever had sex*** | | | | | | | |
| ***Forced into sex during last sexual encounter*** |  |  |  |  |  |  |  |
| ***no*** | 9.1% (71/780) | Reference | <0.0001 | Reference | 0.019 | Reference | 0.045 |
| ***yes*** | 22.9% (16/70) | 2.96(1.61-5.44) |  | 2.14(1.13-4.03) |  | 1.95(1.02-3.74) |  |
| ***Condom use during last sexual intercourse*** |  |  |  |  |  |  |  |
| ***Not used*** | 12.4% (43/346) | Reference | 0.082 | Reference | 0.12 | - | - |
| ***used*** | 8.7% (44/504) | 0.67(0.43-1.05) |  | 0.70(0.44-1.09) |  | - |  |
| ***Alcohol/drug use during last sexual encounter*** |  |  |  |  |  |  |  |
| ***no*** | 9.4% (71/755) | Reference | 0.026 | Reference | 0.004 | Reference | 0.005 |
| ***yes*** | 16.8% (16/95) | 1.95(1.08-3.52) |  | 2.46(1.33-4.54) |  | 2.47(1.32-4.62) |  |
| ***Amongst females*** | | | | | | | |
| ***Currently Pregnant*** |  |  |  |  |  |  |  |
| ***no*** | 7.9% (99/1254) | Reference | 0.1 | Reference | 0.072 | - | - |
| ***yes*** | 16.2% (6/37) | 2.26(0.92-5.54) |  | 2.30(0.93-5.70) |  | - |  |

**Note:**

**†** P-values from Likelihood ratio test

%(n/N) = proportion with depressive symptoms expressed as a percentage (Number with depressive symptoms/denominator)

“-” Information missing

OR = Odds Ratio; AOR = Adjusted Odds Ratio; CI = Confidence Interval;

* For *TB status*; the symptomatic and on treatment were collapsed into one category for the analysis at this stage

Adjusted model 2 = Final model for the main analysis as described on the methods section in the main text
